# Oxycodone at the Crossroads of Pain Management, Misuse, and Toxicological Investigation

**DOI:** 10.3390/metabo16100719

**Published:** 2026-09-28

**Authors:** Dawid Ślebioda, Marta Alicja Ślęczka, Izabela Skowron, Małgorzata Stanclik, Magdalena Mikłuszka, Agnieszka Chłopaś-Konowałek

**Affiliations:** 1 Scientific Society for Neurotoxicology, Department of Forensic Medicine, Wroclaw Medical University, Mikulicza-Radeckiego 4J, 50-345 Wroclaw, Poland; dawid.slebioda@student.umw.edu.pl (D.Ś.); marta.sleczka@student.umw.edu.pl (M.A.Ś.); izabela.skowron@student.umw.edu.pl (I.S.); malgorzata.stanclik@student.umw.edu.pl (M.S.);; 2 Department of Forensic Medicine, Division of Molecular Techniques, Wroclaw Medical University, Sklodowskiej-Curie 52, 50-369 Wroclaw, Poland

**Keywords:** opioid epidemic, opioid crisis, oxycodone, pain management, toxicology, analytical methods

## Abstract

Background: Since the 1990s, the United States has been facing the recurring problem of widespread misuse, addiction and overdoses involving opioid compounds. This phenomenon is known as the opioid crisis or the opioid epidemic. Every day, around 200 Americans die due to opioid use. Nowadays the country is facing the fourth wave, dominated by fentanyl combined with stimulants. The problem of opioid abuse is not confined to the United States alone, but is a global issue. Objective: In this paper, we do not focus on fentanyl, but on another painkiller, oxycodone, as it is often overlooked in discussions of the current crisis, whilst its sale and use are becoming increasingly common. In Poland alone, the number of prescriptions increased by 50% between 2019 and 2023, and nearly 200 Poles have died as a result of an opioid use disorder. This narrative review examines oxycodone at the intersection of pain management, non-medical use, and toxicological investigation, with emphasis on its pharmacology, clinical applications, abuse liability, overdose risk, and detection in biological matrices. Methods: PubMed and Google Scholar were searched using terms related to oxycodone, toxicology, addiction, the opioid crisis, detection methods, biological matrices, analgesic effects, pharmacokinetics, metabolism, overdose, and hospital treatment. English- and Polish-language publications from January 2020 to July 2026 were prioritised, with the search extended to 2000 where necessary. No formal systematic-review protocol was applied. Results: By interacting with opioid receptors, oxycodone relieves moderate to severe pain. It is therefore used as an analgesic for various types of pain—intraoperative, postsurgical or chronic pain, including cancer-related pain. It has good bioavailability following oral administration and accumulates in tissue less than, for example, morphine. A further advantage is the availability of a variety of routes of administration: in addition to oral administration (including through immediate- and extended-release formulations), oxycodone can also be administered, inter alios, intravenously, intranasally, and rectally. On the other hand, oxycodone causes a range of adverse effects—it can lead to respiratory depression and constipation and has high potential of addiction. Its use is associated with a risk of developing tolerance, and discontinuation may trigger withdrawal symptoms. Oxycodone’s high utility for medical purposes makes it a widely used drug in hospital settings. At the same time, there is an increasing number of cases of inappropriate oxycodone use. This results in the need for analytical methods to monitor concentrations in hospitalised patients and to investigate concentrations in cases of suspected poisoning. The toxicological methods used for detection of oxycodone include both screening tests, such as immunoenzymatic assays, and confirmatory tests—i.e., liquid chromatography/mass spectrometry. The process of choosing an appropriate method begins at the stage of selecting the diagnostic sample. Both standard samples, such as blood (including dried blood spots—a novelty) and urine, and alternative samples—oral fluid, hair, nails and vitreous humour—are useful, depending on the patient’s condition, their availability and the time elapsed since the substance was ingested. Conclusions: Oxycodone remains an important analgesic but carries substantial risks of misuse, addiction, and fatal toxicity. Safe prescribing and monitoring, recognition of drug interactions and individual variability, and integrated interpretation of individual case circumstances, appropriate biological samples, and confirmatory mass-spectrometric findings are essential in clinical and forensic practice.

## 1. Introduction

For over 30 years, the United States of America (USA) has been struggling with the problem of inappropriate opioid use and abuse, including numerous cases of addiction and fatalities resulting from overdoses [1,2]. In 2020 alone, opioids were responsible for approximately 70,000 deaths [3]. By comparison, fewer than 50,000 adults died from alcohol-induced causes in the same year. These causes include alcohol-induced pseudo-Cushing’s syndrome, mental and behavioural disorders due to use of alcohol, degeneration of the nervous system due to alcohol, alcoholic polyneuropathy, alcoholic myopathy, alcoholic cardiomyopathy, alcoholic gastritis, alcoholic liver disease, alcohol-induced acute and chronic pancreatitis, alcohol in the blood, and poisoning by alcohol accidentally, intentionally or by undetermined intent [4]. Nearly 200 people in the USA die from opioid-related causes every day [5].

This phenomenon is known as the opioid crisis or opioid epidemic. The first wave was observed as early as the 1990s and primarily involved mass overdoses of prescription drugs. Pharmaceutical companies assured doctors that the risk of addiction to new painkillers (e.g., OxyContin) was minimal, which encouraged doctors to prescribe them. Due to racial disparities in healthcare and limited access to opioid painkillers (among Black and Latino populations), overdose mortality rates during the first wave were higher among non-Hispanic whites and people aged 45–54 [6].

The second wave began in 2010. At that time, significant restrictions were placed on the prescribing of medications, and the Food and Drug Administration (FDA) approved a reformulated, abuse-deterrent version of OxyContin—which reduced the non-medical use of the drug by as much as 40%. However, there was also an unintentional effect of the increased abuse of substitute drugs, primarily heroin (it is estimated that between 2010 and 2013, as much as 80% of the threefold increase in mortality due to heroin overdose may have been caused by the reformulation of OxyContin) [7]. The overdose curve then shifted to younger age groups. During the first two waves, heroin use increased among non-Hispanic whites but decreased among non-white groups [6].

The third wave occurred in 2013 and involved the use of synthetic opioids, particularly fentanyl [8]. Between 2013 and 2014, deaths from synthetic opioid overdoses (excluding methadone) increased by 79% in the USA—a trend strongly correlated with the spread of fentanyl use [9]. Mortality rates during this phase were highest among white populations [6].

Currently, there is already talk of a fourth wave, in which illegal fentanyl taken in combination with stimulants (primarily methamphetamine and cocaine) is leading the way, a practice that is becoming increasingly common [6]. Other opioids most commonly used during the current wave include hydrocodone, hydromorphone, morphine and oxycodone [10].

This phenomenon is not limited to the USA. The opioid crisis is global in scale. In Canada, between 2016 and 2022, more than 32,000 people were hospitalised, and nearly 31,000 died as a result of opioid overdoses [11]. In Lebanon and Libya, opioids account for 90% of drug overdose deaths [12]. According to the 2026 edition of Australia’s Annual Overdose Report by the Penington Institute, in Australia, almost half of all drug-related deaths are caused by opioids—this number (877 deaths) is lower than the peak of deaths in 2018 (1101 deaths), but higher than in the previous year (806 deaths) [13].

Meanwhile, Europe is not facing as much of a growing issue as other parts of the world, particularly the USA [1,14]. Mortality, even despite an increase in the number of opioid prescriptions since 2009, remains steady at 1.2 per 100,000 residents per year [14]. For example, in Poland, in just four years between 2019 and 2023, the number of prescriptions for oxycodone, marketed under the trade name OxyContin—the best-selling opioid drug on the Polish market—increased by about 50%, from over 170,000 in 2019 to about 260,000 in 2023 [15], yet the number of deaths caused by opioid use disorder remains relatively low. In 2019, it stood at just under 200 people [16].

The high number of opioid prescriptions and the inappropriate and excessive use of these medicines are some of the factors contributing to the opioid crisis. A further issue is the poor storage and improper disposal of unused medicines [17]. This is a particular problem in the context of home care. When medicines are left unsupervised, this increases the risk of them being ingested by the wrong people, including children and teenagers, which poses a risk of subsequent addiction or poisoning. Among children and young people, the number of deaths from accidental opioid poisoning ranges from 0.1 to 3.7 per 100,000 people [18]. Current recommendations on the disposal of medicines in the USA advise that leftover medicines should be handed in to special programmes set up for the collection and disposal of medicines. These are usually located in hospitals, pharmacies or police stations [19]. In addition to these regular programmes, a series of temporary events have been organised, the so-called National Prescription Drug Take Back [17]. If the designated disposal schemes are not available, unused opioids may be flushed down the toilet. Recommendations regarding this method vary by region, as it poses a risk of environmental contamination [19]. Unused medicines can also be disposed of in the bin if they have first been mixed with non-edible substances, such as cat litter and coffee grounds [17].

Fentanyl is the primary compound associated with the fourth wave; however, in this article we will focus on analysing the role of oxycodone, as current research tends to focus less on this substance amidst the rising tide of fentanyl poisonings. However, oxycodone—thanks to its good bioavailability, sufficient potency to relieve even severe pain, and wide range of administration routes—is a common choice as a pain-relief medication in hospital settings.

Oxycodone is a potent semisynthetic analogue of naturally occurring thebaine (an opium poppy alkaloid) [20]. It belongs to the phenanthrene class of opioids (along with morphine and codeine) [21]. It is indicated for the treatment of pain, including cancer pain, post-operative pain, pain associated with rheumatic conditions, and neuropathic pain [22]. Additionally, it produces relaxation and anxiolysis.

In the early 20th century, when heroin and morphine were abused on a large scale, scientists wanted to synthesise a new, powerful, non-addictive opioid for use as a pain reliever [23]. In 1916, Martin Freund and Edmund Speye [24] synthesised oxycodone in Germany by modifying thebaine [25], and it was introduced into clinical use in that country a year later [23]. During World War II, it was widely used for its analgesic and sedative effects [26]. However, as early as the 1940s, concerns arose that oxycodone might have a significant addictive potential similar to that of heroin and morphine [23]. Formal approval from the FDA was granted in 1991. In 1995, oxycodone became available in an extended-release (ER) formulation, and in 1996 as an immediate-release (IR) formulation and a single-dose product (SDP) [23]. A significant moment was Purdue Pharma’s introduction of an ER oxycodone formulation called OxyContin in 1996. A crucial part of the promotional campaign was the assurance that the risk of addiction to OxyContin was extremely low—“less than one percent” [27]—which led doctors to prescribe it willingly. However, it was easy to destroy the controlled-release (CR) formulation (by crushing the tablet) and absorb the substance (e.g., by inhalation or injection), which leads to intense euphoria. These events led to a growing opioid addiction problem, known as the opioid crisis, in which oxycodone has played a significant role [23].

The history of oxycodone is presented in Figure 1.

Although the number of oxycodone-related deaths in the USA has fallen recently (from 1.9 per 100,000 standard population in 2016 to 1.5 per 100,000 standard population in 2021), 5060 people still died from oxycodone poisoning in 2021 alone [28]. In 2020 in Australia, more than half of opioid-related deaths were caused by oxycodone or codeine, exceeding even the number of deaths caused by fentanyl [12]. Almost 800 people suffered from oxycodone poisoning in Switzerland in 2019 [29]. Sweden reports that the number of deaths related to the use of oxycodone stood at 575 between 2006 and 2018 [30]. In France, oxycodone was one of the few opioids to show an increase in its percentage share of deaths caused by painkillers between 2013 and 2022 [31].

However, the high mortality rate does not deter the use of oxycodone in the hospital setting. The number of prescriptions for oxycodone is rising every year [32]. Between 2009 and 2019, oxycodone was the most commonly consumed opioid in the world [33]. Between 1991 and 2019, over 110 million prescriptions for oxycodone were issued [34].

The reason why oxycodone is prescribed on such a large scale is due to its pharmacological and pharmacokinetic properties. It is an extremely useful drug in various fields of medicine. However, its widespread use makes it a compound that is frequently involved in cases of abuse (use of substance to obtain psychotropic effects), misuse (the use of the medicine for purposes other than those for which it is prescribed), addiction (compulsive use) and intoxication (disrupted body function due to xenobiotic intake) [35]. Consequently, there is a need to develop new methods for the detection and analysis of oxycodone in toxicology, which is characterised by higher sensitivity and specificity, as well as lower limits of detection. The choice of test sample plays a major role here—ranging from traditional samples such as blood or urine to alternative ones such as hair or oral fluid. Each has a different detection time following ingestion of the compound, different advantages and disadvantages, and different stabilities over time, making them suitable for different purposes and in different situations.

## 2. Methods

This article has been written as a narrative review focusing on the topic of oxycodone—its physicochemical properties, its role in the medical field, and the risks associated with the use of this drug, particularly in the context of misuse and addiction. Furthermore, we expand on previous articles published on this topic, including those by Barrett et al. (2021) [23] and Kibaly et al. (2020) [20], by adding a comparison of various matrices and methods for detecting oxycodone in toxicology, with the aim of identifying the best method for detecting this compound depending on the clinical situation and the materials available.

In our study, we used PubMed and Google Scholar to search for articles containing keywords such as, amongst others, ‘oxycodone’, ‘toxicology’, ‘addiction’, ‘opioid crisis’, and ‘opioid epidemic’, and combinations of keywords, one of which was usually ‘oxycodone’, combined with, for example, words such as ‘detection’, ‘matrix’, ‘hospital’, ‘treatment’, ‘analgesic effect’, ‘pharmacokinetics’, ‘metabolism’, ‘addiction’ and ‘overdose’. Boolean operators AND and OR were used to combine the search terms.

We primarily referred to articles available in Polish and English published between January 2020 and July 2026. Where necessary, we extended the scope of our search back to the year 2000.

As this is a narrative review, we did not use any formal review protocol, such as PRISMA, but simply sought to present the content and the issue in the most accessible way possible, whilst adhering to all standards.

## 3. Results

### 3.1. The Pharmacokinetics of Oxycodone

#### 3.1.1. Chemical Structure

Oxycodone (14-hydroxy-7,8-dihydrocodeinone, or, to give it its full name, 4,5α-epoxy-14-hydroxy-3-methoxy-17-methylmorphinan-6-one hydrochloride) [36] (shown in Figure 2) belongs to the opioid class, i.e., compounds that act on opioid receptors (a more detailed description is presented in Section 3.1.10). Its structure resembles hydrocodone, but it possesses a hydroxyl group at the 14th carbon atom [21]. It is a lipophilic compound with a low molecular weight (315.36 g/mol). It has four chiral centres [36]. It has a methoxy group at position 3, which prevents direct glucuronidation in the liver, thereby preventing rapid inactivation of the compound and contributing to the high bioavailability [22].

In the USA oxycodone is used as a hydrochloride or terephthalate salt to increase its absorption in the digestive system [37].

#### 3.1.2. Route of Administration

Oxycodone can be administered in several different forms, including orally, rectally, intravenously, intramuscularly [38], and epidurally. It is worth noting that, regardless of the route of administration, the pharmacokinetics of oxycodone are dose-independent. The pharmacokinetics or pharmacodynamics of oxycodone are not significantly affected by taking the substance with food. Interestingly, in the context of analgesia, it has been shown that epidural administration of oxycodone produces a stronger effect than intravenous administration of the substance [39,40]. The FDA has approved IR, ER, and abuse-deterrent formulations of oxycodone containing inactive ingredients [38]. IR tablets are available in 5 mg, 10 mg, 15 mg, 20 mg, and 30 mg, and capsules are available in 5 mg. An oral solution (5 mg/5 mL) and concentrate (100 mg/5 mL) are also accessible. ER tablets must not be broken, crushed, chewed, or dissolved. They are available in doses of 10 mg, 15 mg, 20 mg, 30 mg, 40 mg, 60 mg, and 80 mg. Oxycodone abuse-deterrent tablets are manufactured in doses of 9 mg, 13.5 mg, 18 mg, 27 mg, and 36 mg. Oxycodone is also available in combination with other analgesics, including aspirin [38].

#### 3.1.3. Bioavailability

Compared to morphine (the archetypal opioid), oxycodone exhibits higher oral bioavailability (60–87%) because it undergoes first-pass metabolism to a lesser extent than morphine. For rectal administration, bioavailability is 50–60%, and for intranasal administration, it can depend on the patient [22]. In contrast, it exhibits low bioavailability when administered sublingually [39].

#### 3.1.4. Absorption

CR oxycodone formulation consists of two acrylic polymers; consequently, its absorption occurs in two phases. The first phase begins within an hour and involves erosion of the tablet surface, allowing rapid release and dissolution of oxycodone. The half-life of the first phase is 0.6 h and accounts for around 38% of the dose. This is followed by the ER phase, which can last a maximum of 12 h. Its half-life is 6.9 h and accounts for the remaining 62% of the dose [22]. Steady-state plasma concentrations of oxycodone are reached within 24–36 h, and the plasma half-life, which is not affected by the route of administration, is 3–5 h [38]. It is worth highlighting that oxycodone absorption tends to be more pronounced in female patients compared to males, and it progressively scales with advancing age [39]. Additionally, due to slower drug elimination in women, plasma concentrations of oxycodone are slightly higher in this population [22].

#### 3.1.5. Membrane Permeability

Oxycodone easily diffuses through intercellular spaces and enters the cell interior [22]. Oxycodone also crosses the blood–brain barrier, most likely via active transport, which contributes to the rapid onset of action of this substance. The onset of analgesic action following intravenous administration is observed as early as approximately 6 min [26]. Other sources report that the onset of action of oxycodone occurs after 10–30 min for IR formulations and after approximately 1 h for CR formulations [38]. This time is shorter than with morphine administration. The metabolites of oxycodone (oxymorphone and noroxycodone) have a significantly lower, even limited, ability to cross the blood–brain barrier. Oxycodone, being an opioid, can also cross the placental barrier [38].

#### 3.1.6. Distribution and Duration of Action

The volume of distribution of oxycodone following intravenous administration is 2.6 L/kg [36]. It is widely distributed to the central nervous system and certain peripheral tissues, including the skeletal muscle, liver, gastrointestinal tract, lungs, and spleen [41]. The duration of action of oxycodone depends on the formulation. With IR formulations, the duration of action is 3–6 h, whereas for CR formulations, it is longer, lasting 12 h. Between 38% and 45% of oxycodone is bound to plasma proteins, primarily albumin, meaning that up to 62% of the compound may be pharmacologically active [38].

#### 3.1.7. Metabolism

Oxycodone is metabolised in the liver via several processes: O-demethylation catalysed by the cytochrome P450 isoenzyme CYP2D6, resulting in the formation of oxymorphone (this accounts for approximately 19% of the total oxycodone dose), and N-demethylation via the CYP3A4/5 isoenzyme, which leads to the formation of noroxycodone (approximately 45% of the total dose of oxycodone undergoes this metabolic pathway). This is followed by conjugation with glucuronic acid, catalysed by the enzyme uridine diphosphoglucuronosyltransferase (UGT)—UGT2B7 and UGT2B4 [39]. This results in compounds that are highly soluble in water. It should be noted that a small proportion of oxycodone undergoes 6-ketoreduction to form 6-oxycodol [22] and some of the oxycodone is conjugated with glucuronide, but due to the presence of a methoxy group at position 3, this does not occur rapidly [42].

Variants in the genes encoding the enzymes involved in the metabolism of oxycodone affect the function of these enzymes, which in turn influences the metabolism of oxycodone. Among the CYP3A4 variants, the four of greatest clinical significance are *1b, *2, *3 and *22, and none of these is a non-functional allele. The most common CYP3A5 variant is CYP3A5*3—a non-functional allele found in 85% of people of Caucasian descent and 65% of those of Asian descent. The high degree of genetic variation within the CYP2D6 gene makes it clinically significant. Cases of gene duplication, deletion or overexpression do occur. Some alleles (e.g., *1 and *2) exhibit normal activity, whilst others (e.g., *3 and *4) are inactive. Genetic variations lead to differences in the activity and function of these enzymes. This may result in reduced metabolism and prolong the elimination half-life of oxycodone, thereby increasing exposure to the xenobiotic and raising the risk of adverse effects and hypersensitivity reactions [43].

Oxymorphone is a potent analgesic even at very low concentrations. It has 10 to 45 times greater affinity for µ-opioid receptors than oxycodone, making it 8 to 30 times more potent [36,39]. This compound does not affect the activity of the CYP2D6 and CYP3A4/5 isoenzymes; as a result, it may be a good option for patients taking multiple medicines, as it reduces the number of potential drug interactions. However, as oxymorphone does not readily cross the blood–brain barrier, an oral dose approximately twice as high is required to achieve an analgesic effect comparable to that of oxycodone [36].

Noroxycodone, on the other hand, exhibits mild analgesic effects. It has weak antinociceptive potency and displays one-third the affinity for µ-opioid receptors compared with oxycodone. Consequently, its potency is five to ten times weaker than that of oxycodone [36].

Subsequently, both oxymorphone and noroxycodone are metabolised to noroxymorphone, which, due to its higher affinity for µ-opioid receptors (2 to 4 times greater), exhibits an effect twice as potent as that of oxycodone [36].

The metabolism of oxycodone is presented in Figure 3.

#### 3.1.8. Elimination

Oxycodone and its metabolites are excreted by the kidneys in the urine—clearance following intravenous administration is 0.82 L/min, while following intramuscular administration it is 0.78 L/min [36]. Within the first 24 h, about 8% to 14% of free and conjugated oxycodone is eliminated [22]. Oxycodone is excreted unchanged, as oxidative metabolites (93% of oxymorphone is excreted as a glucuronide, while noroxycodone is mostly unconjugated), and as reduced metabolites [39].

Impaired liver and kidney function reduces clearance. Additionally, the patient’s age also affects oxycodone clearance, which contributes to drug exposure. As the patient ages, oxycodone clearance decreases, prolonging the half-life. Paediatric patients have a 20–40% increase in oxycodone clearance compared to adult patients [39]. Neonates are the exception here, as oxycodone clearance increases with age during the first 6 months after birth, and only then does it start to decline with age. Higher clearance has also been observed in pregnant women. Oxycodone is also excreted into breast milk during lactation [38]. Oxycodone is slowly released from tissues, as a large portion of the compound remains outside the vascular space and is not eliminated by the kidneys or liver.

#### 3.1.9. Receptor Interactions

Oxycodone belongs to the class of opioids, i.e., compounds that act on opioid receptors. Its binding affinity for these receptors is relatively weak compared to morphine, ranging from 1/10 to 1/40 of morphine’s affinity [22]. These are G protein-coupled receptors (GPCRs). They consist of seven transmembrane domains (helices) that bind to intracellular G proteins (composed of subunits Gα, Gβ, and Gγ) [44]. There are various types of G proteins, including Gs proteins, which have stimulatory activity (they activate adenylate cyclase, which increases cAMP production), and Gi proteins, which inhibit cAMP production. Opioid receptors are coupled to the Gi subtype of proteins [45].

Opioid receptors are classified based on their prototypical agonists, and historically their names corresponded to Greek letters [21]. The primary opioid receptor types include Mu (µ; morphine agonist; MOR; MOP), Kappa (κ; ketocyclazocine agonist; KOR), Delta (δ; delta-alanine-delta-leucine-enkephalin agonist; DOR) and nociceptin (NOR; ORL1). Some studies have also identified zeta (ζ), epsilon (ε), lambda (λ), and iota (ι). The sigma (σ) receptor (N-allylnormetazocine agonist) is no longer classified as an opioid receptor, as it shows no affinity for naloxone [21,46].

Oxycodone acts as an agonist across the μ, κ, and δ opioid receptors. It shows the strongest affinity for μ receptors [38]. The presence of µ receptors has been detected in the brainstem and medial thalamus. They are responsible, among other things, for euphoria, sedation, supraspinal analgesia, respiratory depression, gastrointestinal motility, and physical dependence. κ-receptors are found in the midbrain, primarily in the limbic system, the brainstem, and the spinal cord. δ-receptors are also found in the brain. Opioid receptors are located on the presynaptic terminals of nociceptive C-fibres and A-δ fibres [21].

#### 3.1.10. Mechanism of Action

Upon binding to the receptor, the opioid stimulates the exchange of GDP for GTP in the Gα subunit, leading to the dissociation of the Gβγ subunit from Gα [45]. These subunits then act on various intracellular effector pathways—specifically on ion channels and adenylate cyclase. The Gα subunit binds to adenylate cyclase, suppressing its activity and thereby reducing intracellular levels of the second messenger, cAMP. Meanwhile, the Gβγ subunit, by binding to voltage-gated calcium channels of the N type, inhibits their activity [45]. These channels, located in the presynaptic region of neurons throughout the central and peripheral nervous systems, are responsible for the influx of calcium ions into the presynaptic neuron, which leads to the release of neurotransmitters into the synaptic cleft [44]. Additionally, the Gβγ subunit opens G Protein-Gated Inwardly Rectifying Potassium (GIRK) channels, causing K^+^ ions to flow out of the cell, leading to its hyperpolarization [47]. Consequently, the release of pain-transmitting neurotransmitters is inhibited, which accounts for the analgesic effect of these substances [21].

The mechanism of action is presented in Figure 4.

#### 3.1.11. Drug Interactions

Oxycodone’s interactions with other substances are primarily associated with its metabolism via the cytochrome P450 system. Interactions between substances affect, among other things, the clearance, peak plasma concentration, and pharmacodynamics of oxycodone. Drug interactions are primarily influenced by CYP3A inducers or inhibitors and CYP2D6 inhibitors. The CYP3A4 isoenzyme plays a major role in the metabolism of oxycodone; drugs that alter its activity can cause significant changes in oxycodone clearance, which is associated with changes in its plasma concentration. For example, the use of antibiotics (clarithromycin, telithromycin), antifungals (itraconazole, ketoconazole, voriconazole), and antivirals (ritonavir)—all of which are CYP3A inhibitors—reduces oxycodone clearance, leading to an increase in its maximum plasma concentration and an intensification of its effects. It is also worth noting that certain fruits, including grapefruit, may inhibit CYP3A and thus potentiate the pharmacological effects of oxycodone. The use of CYP2D6 inhibitors (such as quinidine and paroxetine) also affects the metabolism of oxycodone, though to a much lesser extent. It should be noted that the concurrent use of CYP2D6 inhibitors with CYP3A4 inhibitors may result in a more pronounced effect of oxycodone than when interacting with only one inhibitor. These interactions are more significant when oxycodone is administered orally [36].

CYP3A4 inducers such as rifampin, carbamazepine, and phenytoin have the opposite effect—they reduce plasma oxycodone concentrations, thereby diminishing the therapeutic effect of oxycodone [38].

Oxycodone does not just interact with compounds metabolised by CYP enzymes. Benzodiazepines and central nervous system (CNS) depressants, including antipsychotics, alcohol, and other opioids, when administered concomitantly with oxycodone, increase the risk of hypotension, sedation, respiratory depression, and death. Concomitant administration of oxycodone with serotonergic drugs is associated with a risk of serotonin syndrome. It can also be induced by concomitant administration of monoamine oxidase inhibitors. Concomitant administration of oxycodone with muscle relaxants may enhance neuromuscular blockade, which exacerbates respiratory depression [38].

#### 3.1.12. Adverse Effects

Oxycodone is a substance with a high potential for addiction. It is classified as a Schedule II substance by the Drug Enforcement Administration and as a Group I-N narcotic by the Polish Ministry of Health [21,48]. Since it is classified as an opioid, it has similar side effects to the rest of the compounds in this group.

Typical side effects include constipation (occurring in 25–30% of cases) [22]. This is caused by inhibition of gastric emptying and reduced peristalsis in the gastrointestinal tract, which leads to increased fluid absorption. This leads to a lack of fluid in the intestines, resulting in hard stools and constipation. Additionally, opioids increase anal sphincter tone, which impairs the defecation reflex [49]. Other gastrointestinal symptoms may also occur, including dry mouth, nausea (occurring in 25%), vomiting (occurring in 10–15%), abdominal pain, diarrhoea, and glossitis. Pruritus (occurring in 10–15%) and hyperhidrosis may also occur. Neurological disorders associated with taking this compound include dizziness (occurring in 15%) and headaches, excessive drowsiness (occurring in 25%), confusion, hallucinations, increased intracranial pressure, irritability, sedation, seizures, and photosensitivity.

This compound also affects the cardiovascular system, causing bradycardia, hypotension, and palpitations. In the respiratory system, respiratory depression and coughing may occur [38]. The severity and frequency of these side effects decrease over time [22].

It is also worth noting the effect of oxycodone on hormonal disorders in the body. Since oxycodone acts as an agonist on opioid receptors located in the hypothalamus, it inhibits the pulsatile secretion of liberin, which in turn inhibits stimulation of the anterior pituitary lobe. This leads to a reduction in the secretion of gonadotropins—FSH and LH—which stimulate the testes and ovaries. Consequently, testosterone levels in men and oestrogen levels in women decrease. This can lead to reduced libido, infertility, and erectile dysfunction [50]. Chronic deficiency of sex hormones during long-term use of oxycodone may contribute to low bone mineral density [51]. What is more, oxycodone negatively impacts the hypothalamic–pituitary–adrenal axis—it inhibits the secretion of corticotropin-releasing hormone, which results in reduced secretion of adrenocorticotropic hormone, leading to decreased cortisol secretion [51]. Oxycodone may also increase serum prolactin levels (most likely by inhibiting the dopaminergic system), which may lead to galactorrhoea [22].

### 3.2. The Medical Application of Oxycodone

The primary site of action of oxycodone’s analgesic effect is the central nervous system, comprising the brain and spinal cord. The substance also exhibits peripheral action to a certain degree, which may play a role in pain modulation, particularly in the context of inflammation and visceral pain [36].

Oxycodone is a medicine used to treat pain following injuries, including bone injuries, after surgeries [20], and also in the case of pain associated with cancer [52]. Its uses also include the treatment of chronic non-cancer-related neuropathic pain [20]. Oxycodone is used for analgesic premedication prior to radiotherapy in patients with cancer-related pain. It has been shown that the administration of oxycodone hydrochloride during radiotherapy effectively prevents breakthrough pain episodes and discomfort associated with changes in body position, thereby allowing treatment to continue uninterrupted. Furthermore, this substance exhibits beneficial analgesic effects in radiation-induced stomatitis, with the best therapeutic results observed in cases of mild pain [53]. This medicine is used to treat moderate to severe pain [54]. However, its effectiveness in the case of diabetic neuropathy may not be sufficient [39,55]. Oxycodone can be used as the sole opioid in general anaesthesia, ensuring haemodynamic stability and effective pain control both during and after minor and moderate surgical procedures [56].

Compared with morphine, oxycodone has fewer side effects due to the lower accumulation of toxic metabolites in tissues, and is therefore more suitable for patients [39].

It has been shown to be highly effective in epidural anaesthesia, which has made it possible to use lower doses of analgesics in subsequent treatment. The latest reports indicate that, compared to fentanyl, oxycodone can effectively alleviate the body’s response to endotracheal intubation and help to maintain stable haemodynamic parameters [57].

In gynaecology, following a caesarean section, oxycodone is used to treat post-operative pain either orally or intravenously. Oral oxycodone is an equivalent alternative to patient-controlled intravenous analgesia for the treatment of post-operative pain following a caesarean section, providing comparable analgesic efficacy and patient satisfaction whilst resulting in a lower incidence of nausea and vomiting [58,59,60].

Oral administration is also around twice as cheap as intravenous patient-controlled analgesia, and its use is associated with a lower risk of developing chronic opioid dependence [60,61]. Given the specific nature of the postnatal period—the need for early mobilisation, the establishment of the bond between mother and child, and breastfeeding—the oral form of oxycodone appears to be a particularly clinically justified treatment option [60,62,63].

Since 2014, oxycodone has been the most commonly prescribed oral painkiller for children in the USA for the treatment of moderate to severe pain [64]. Despite its growing popularity, data on the safety and efficacy of oxycodone in this age group remain limited; therefore, oral morphine is still considered a treatment option with a better-documented clinical profile for the management of pain in children [64,65].

In the treatment of varicose veins and endoscopic injection sclerotherapy (EIS) for oesophageal varices, oxycodone—particularly when combined with midazolam—provides better analgesic effects and is less likely to cause hypoxia than sufentanil [66].

#### 3.2.1. Dosage Forms

Oxycodone can be administered via various routes. It is administered, amongst other routes, intravenously, intramuscularly, intranasally, subcutaneously, rectally, epidurally and orally—both as IR solutions and as IR or CR tablets [43]. Oxycodone is available on the pharmaceutical market in several forms. In oral form it is available both as a single-ingredient preparation and in ER, CR and IR formulations. Oxycodone is also an ingredient in combination preparations, in which it is combined with non-opioid analgesics—such as aspirin or paracetamol [20].

The oral form of oxycodone has demonstrated superior analgesic efficacy compared with placebo in the management of post-operative pain in patients undergoing a variety of surgical procedures, such as laparoscopic cholecystectomy, abdominal and pelvic surgery, bunionectomy, breast surgery and spinal surgery. Compared with intravenous opioids, oral oxycodone achieves equivalent and sometimes even better results in terms of pain relief following knee replacement, spinal surgery, caesarean section, laparoscopic colorectal surgery and cardiac surgery [67].

Oxycodone HCl in the form of prolonged-release tablets is available in two main formulations. The original formulation, launched in 1995 under the name OxyContin^®^ (Purdue Pharma), did not have any abuse-deterrent properties. In response to the growing problem of opioid misuse, in 2010, the formulation was reformulated by incorporating polyethylene oxide, which creates a physical barrier preventing the mechanical crushing of the tablet and the extraction of the active substance using solvents, thereby significantly reducing the possibility of parenteral or intranasal administration of the medicine [68].

The ER tablet works in two stages—immediately after swallowing, it releases around 40% of its active ingredient, which means the first noticeable pain-relieving effect is felt after about an hour; then, it gradually releases the remaining oxycodone over the next 8–12 h, ensuring prolonged pain control.

Oxycodone acts more quickly than morphine, as it crosses the blood–brain barrier more efficiently; as a result, patients experience pain relief after around 30 min, and effective concentrations in the central nervous system are maintained for several hours thereafter [69].

Oral oxycodone provides comparable pain control and patient satisfaction to intravenous patient-controlled analgesia following a caesarean section; both forms are well tolerated, and early nausea is less common with oral administration [60]. Numerous studies have documented that the oral form of oxycodone significantly reduces the need for rescue analgesia and the total consumption of opioids. Patients treated in this way experienced a lower incidence of typical opioid side effects, whilst the incidence of post-operative nausea and vomiting remained comparable to that of the placebo group. Furthermore, oral oxycodone has no adverse effects on the length of hospital stay, whilst resulting in lower costs of pharmacotherapy compared with epidural and intravenous analgesia. The inclusion of oxycodone in a multimodal pain management regimen is associated with better analgesic control, a reduction in the incidence of adverse effects, and a shorter length of hospital stay [67]. The bioavailability of oral oxycodone in patients with short bowel syndrome is clinically significant, but is characterised by considerable inter-individual variability, depending on the length of the remaining small intestine and the presence of the colon. This requires strict individualisation of the dosage, taking into account nutritional status and gastrointestinal motility [70].

Intravenous analgesia is primarily used in the early post-operative period, when oral administration is difficult or impossible. In the case of moderate to severe acute post-operative pain, current clinical guidelines recommend the use of strong opioids—such as oxycodone, morphine, fentanyl or hydromorphone [71,72]. Oxycodone has an analgesic advantage over fentanyl, particularly in the early post-operative period—significantly better pain control is achieved as early as 30 min after surgery and may persist for up to 48 h, although the differences between these two substances disappear in the later post-operative period [71]. Intravenous oxycodone has a comparable incidence of adverse effects such as nausea, vomiting and pruritus to that of morphine, but causes sedation significantly less frequently in the first 24 h following surgery. Like other opioids, it may cause central nervous system depression, gastrointestinal disturbances and respiratory depression, requiring appropriate patient monitoring. Available data suggest that oxycodone causes hallucinations and nightmares less frequently than morphine, which may represent a clinically significant advantage in the post-operative period [73,74,75]. Intravenous administration of oxycodone immediately after the end of surgery significantly stabilises haemodynamic parameters—blood pressure and heart rate—both during and in the first 10 min following extubation [76].

In the treatment of chronic pain, the oral route of administration of analgesics is considered the most optimal in terms of convenience and therapeutic efficacy. However, in certain clinical situations, such as dysphagia, nausea, vomiting or gastrointestinal obstruction, this route of administration is not possible. In such cases, the intranasal route of administration of oxycodone represents a potential therapeutic alternative [77].

Oxycodone is characterised by rapid and relatively effective absorption through the nasal mucosa, although the bioavailability of this route of administration is characterised by considerable inter-individual variability. The oral ER formulation is characterised by rapid absorption through the nasal mucosa, which may be an advantage in certain clinical indications; however, the abuse-resistant prolonged-release oxycodone hydrochloride causes significant discomfort and irritation of the mucous membrane when administered via this route, which significantly limits its therapeutic usefulness in intranasal use, whilst the safety profiles of both formulations remain comparable. The intranasal form of oxycodone may represent a valuable alternative to oral or parenteral administration in selected clinical situations; however, due to the aforementioned pharmacokinetic variability, individualised dosing remains a key element of safe and effective therapy [77,78,79].

Epidural administration of oxycodone is a valid therapeutic option, given that the central nervous system is the primary site of action for opioid analgesia. Epidural administration allows a higher concentration of oxycodone to be achieved in the CNS compared with an equivalent intravenous dose, which translates into a potentially better analgesic effect [80,81]. A total of 60% of epidural oxycodone crosses into the cerebrospinal fluid, whilst 40% enters the bloodstream [80]. The epidural route of administration of oxycodone results in significantly higher concentrations of the active substance in the cerebrospinal fluid, translating into potentially better analgesic efficacy compared with the intravenous form [40]. Epidural administration of oxycodone represents a valuable therapeutic option in both these situations—in labour analgesia, its use in combination with a local anaesthetic enables effective control of labour pain whilst reducing the dose of the anaesthetic, thereby eliminating the risk of motor block. In the management of post-operative pain following extensive abdominal and thoracic surgery, epidural oxycodone, often administered in combination with local anaesthetics, clonidine or adrenaline, provides effective analgesia, alleviates the stress response to surgical trauma and reduces the risk of complications such as respiratory failure, ileus or delirium [82,83,84,85]. Epidural administration of oxycodone is an effective analgesic option for the management of post-operative pain following gynaecological laparoscopy, providing better pain control than the intravenous route. Oxycodone administered via this route is particularly useful as part of multimodal pain management, significantly reducing the need for additional rescue analgesics in the first few hours after surgery [86].

Oxycodone can also be administered rectally, which represents an important therapeutic alternative in cases where oral administration is impossible or contraindicated. This route bypasses the portal circulation, eliminating the first-pass effect in the liver, which results in higher blood concentrations of the drug. Furthermore, it prevents the active substance from coming into contact with the acidic environment of the stomach and digestive enzymes, thereby protecting the drug from degradation, minimising irritation of the gastrointestinal mucosa and significantly increasing its bioavailability [87]. A summary of the various routes of administration for oxycodone is set out in Table 1.

#### 3.2.2. Oxycodone–Naloxone Formulation

Opioid-induced constipation is one of the most common and most troublesome adverse effects of long-term opioid therapy in patients with cancer pain. In response to this problem, a fixed-dose combination of oxycodone and naloxone in the form of prolonged-release tablets has been developed, which combines the analgesic effects of the opioid with peripheral µ-receptor antagonism. The prolonged-release fixed-dose combination of oxycodone and naloxone is available on the pharmaceutical market under trade names including Targin^®^, Targinact^®^ and Targiniq^®^. This is a medicine taken every 12 h, authorised in European Union countries for the treatment of severe pain in adults requiring opioid analgesics [88]. The oxycodone and naloxone formulation is used in a fixed 2:1 dose ratio, which ensures optimal and consistent analgesic effects whilst alleviating opioid-induced constipation. Naloxone, characterised by low oral bioavailability and extensive first-pass metabolism, acts mainly locally in the gastrointestinal tract without causing significant systemic effects, although diarrhoea may occur temporarily at the start of treatment. The prolonged-release formulation provides a gradual release of both active substances over an extended period, ensuring sustained analgesia, reducing the frequency of dosing and improving patient adherence to treatment regimens [89,90,91]. Naloxone, by acting selectively on opioid receptors in the gastrointestinal tract, counteracts the adverse intestinal effects of oxycodone whilst preserving its central analgesic effect [92,93]. The concomitant use of oral ER naloxone in combination with oral ER oxycodone leads to a significant improvement in bowel function compared with oxycodone monotherapy, without adversely affecting its analgesic efficacy [94]. Both components of the combination preparation are available in an oral, prolonged-release formulation. Naloxone acts locally within the gastrointestinal tract, antagonising the inhibitory effect of the opioid on intestinal motility; however, once absorbed, it is almost completely inactivated as a result of extensive first-pass metabolism in the liver, which eliminates its central antagonistic effect [95]. The ER formulation of oxycodone with naloxone is strictly contraindicated in patients with moderate to severe hepatic impairment; however, particular clinical caution is required when using it in patients with mild hepatic impairment or renal impairment and the elderly [88]. The combination of oxycodone and naloxone in a single formulation offers a solution aimed not only at pain management, but also at minimising the key limitations of long-term opioid therapy. The presence of naloxone not only counteracts the peripheral adverse effects of oxycodone but also—by inhibiting microglial activation and antagonising the toll-like receptor 4 (TLR4)—may limit the development of opioid tolerance, thereby reducing the need for gradual dose escalation to maintain effective analgesia [96,97,98,99].

### 3.3. The Non-Medical Use of Oxycodone

Non-medical use of oxycodone involves consuming the drug for recreational reasons (e.g., to experience euphoria) or using someone else’s prescription [20,100,101]. In 2014, 10.3 million people in the USA reported non-medical prescription-opioid use, underscoring that this is a large population-level problem rather than a rare behaviour [102]. Several years later, in 2021, data from the National Survey on Drug Use and Health showed that as many as 8.3 million Americans still were misusing opioids [103].

It has been established that oxycodone carries extreme risks, including the rapid development of tolerance (a condition in which the body becomes accustomed to the use of a medicine, thereby requiring an increase in the dose to achieve the desired effect), opioid use disorder (addiction) [104], severe respiratory depression [105,106,107] and fatal overdose [20].

Non-medical oxycodone use commonly includes route alteration. Oxycodone products are crushed for immediate burst release, then snorted or dissolved for injection [20]. In treatment-assessed populations, non-oral use is common for oxycodone products, and snorting and injection remain prominent routes, especially for other ER and IR formulations [108,109].

Route alteration matters because faster delivery increases reinforcing and euphoric effects and is associated with more severe use disorder and overdose risk [110]. Samples taken from rural areas also indicate high rates of snorting OxyContin and oxycodone, with the concern that tampering of slow-release products compounds overdose danger [111].

Oxycodone has unusually high abuse liability in the prescription-opioid class. Survey data cited in a mechanistic review found ER oxycodone was the most liked and wanted drug for euphoria in about half of prescription-opioid-dependent patients. Users often describe oxycodone as producing a particularly smooth and enjoyable high [20].

That subjective appeal has a biological basis in reward circuitry. Oxycodone increases nucleus accumbens dopamine in animal models, and this dopamine rise is paired with respiratory suppression [112]. Mesolimbic dopamine D_3_ receptors are critically involved in oxycodone reward, because deleting D_3_ receptors reduced oxycodone self-administration and oxycodone-enhanced brain-stimulation reward in mice [113].

Addiction risk also persists after stopping the use, because craving can incubate during abstinence. In rodent models, oxycodone seeking intensifies during withdrawal and depends on plasticity in nucleus accumbens AMPA-receptor signalling [114,115]. Abstinence is also accompanied by dysfunctional dopamine uptake in the nucleus accumbens, a change linked to sustained oxycodone seeking [116].

Opioid reinforcement is not only a classic opioid-receptor phenomenon. Preclinical work indicates innate immune signalling through TLR4 can amplify opioid-induced dopamine release and reward, and oxycodone shows altered signalling in TLR4-knockout mice [117].

Respiratory depression is the proximate cause of death in opioid overdose [106,107,118]. Oxycodone is a major contributor to overdose mortality in the USA, with more than 33,000 oxycodone-involved deaths reported from 2011 to 2016 in one review [20].

The danger increases with dose escalation and rapid-onset administration routes. Reviews of opioid toxicology note that high doses and rapid delivery are especially dangerous because they more abruptly depress breathing and brain oxygenation [119]. Non-oral prescription-opioid exposure is also linked to worse acute outcomes than oral exposure, with a reported relative risk of life-threatening outcome or death of 2.43 for non-oral versus oral opioid exposure [120].

Polysubstance use sharply worsens overdose risk. Oxycodone deaths often involve synergy with alcohol, benzodiazepines, or other sedatives [20]. Benzodiazepines and opioids have different pharmacologies, but combined effects that produce significant respiratory depression [121]. More broadly, a 2025 meta-analysis identified benzodiazepine use, problematic alcohol use, and polysubstance use as risk factors for non-fatal opioid overdose [122].

Young opioid-using cohorts show the same pattern. In New York City, most recent overdoses were usually polysubstance events, and benzodiazepine use and prescription-opioid injection were independent correlates of lifetime overdose [123]. Naloxone remains central to overdose response, but reversal is less complete when opioids are co-ingested with ethanol, benzodiazepines, antidepressants, or other central depressants [124].

Repeated opioid exposure can produce physical dependence within days to weeks [125]. With longer use, tolerance and physical dependence are expected, and tolerance means that increasingly larger amounts are needed to achieve the same desired effect [125,126].

Withdrawal is physiologically and psychologically severe. Reviews describe aches, muscle spasms or cramps, nausea, vomiting, diarrhoea, anxiety, insomnia, sweating, and other adverse effects after discontinuation [126]. Withdrawal symptoms are a major driver of relapse during early discontinuation [125].

Oxycodone-specific preclinical studies support this pattern. After stopping chronic oxycodone, mice show increased somatic withdrawal signs, mechanical and cold hypersensitivity lasting days to weeks, and later anxiety-like behaviours [127]. Another mouse model found chronic oxycodone exposure produced opioid-induced hyperalgesia that persisted for up to 7 days after discontinuation, alongside emotional and motivational deficits during withdrawal [128].

Dependence is not identical to addiction, but it increases vulnerability. Reviews distinguish physiological dependence from addiction while emphasising that withdrawal and negative effects can help to maintain compulsive opioid use [126,129]. In chronic pain cohorts, tolerance, interdose withdrawal, and craving predict more severe prescription-opioid use disorder over time [130].

Injection introduces a second major hazard class: infectious disease. Injection-drug use is associated with rising HIV and hepatitis C infections [6]. Hepatitis C has been described as a hidden danger of the opioid epidemic, specifically because of spread through injection-opioid use [131].

Clinical cohorts show how large that burden is. Among USA veterans with opioid use disorder, people with a history of injection-drug use had 62% HCV prevalence, 4.2% HIV prevalence, and 16.2% severe injection-related infections [132]. In young adult PWID (People Who Inject Drugs) in rural New Mexico, 60.9% had HCV exposure, and polysubstance injecting was associated with substantially higher HCV prevalence [133].

In an Australian PWID cohort, the commonest injected drugs were opioids, 46.5% had active HCV infection, and over half required surgical intervention for skin, soft-tissue, or joint infections [134].

Endocarditis is a particularly serious complication. National estimates suggest USA hospital admissions for injection-related endocarditis rose from about 2900 in 2013 to more than 20,000 in 2017 [135]. Another national analysis found the proportion of infective endocarditis hospitalizations linked to injection drug use increased from 7% to 12.1% between 2000 and 2013 [136].

Non-medical prescription-opioid use can also progress into riskier opioid trajectories. National and veteran cohort data show non-medical prescription-opioid use is strongly associated with later heroin initiation [102,137]. Young adult trajectory studies further suggest progression from non-medical IR oxycodone or hydrocodone use to heroin, and injection often occurs within a few years [138].

The non-medical use of oxycodone has significantly influenced the global opioid crisis, with growing rates of addiction and related public health concerns [26]. Previous research showed that this issue is further deepened by high abuse potential [139].

#### 3.3.1. The Mechanism of Oxycodone Addiction

Previous studies have shown that addiction is an inability to control drug intake regardless of negative consequences, which reflects the complex interplay of both psychological and neurobiological factors [140].

Oxycodone exhibits favourable pharmacokinetic properties, including higher bioavailability than morphine, improved CNS penetration, a longer half-life with active metabolite formation, absence of toxic metabolites in patients with kidney disease, and reduced histamine release. These characteristics contribute to its preference in various prescribing scenarios [101,105,141,142].

It has been established that repeated oxycodone intake often results in neuroadaptations, including tolerance and dependence, which can subsequently increase the use of more potent opioids, resulting in severe neuropsychiatric and neuropathological complications [143,144].

Literature review shows that it is crucial to understand the neurobiological mechanism of oxycodone addiction for predicting vulnerability and preventing the progression of this chronic brain disease [145].

A series of studies have indicated that oxycodone, like other opioids, primarily utilises its effects by binding to G-protein-coupled opioid receptors located in the central and peripheral nervous systems, with apparent effects observed in the dorsolateral striatum, including conditioning behaviour and neuronal activity [146].

Oxycodone has a complex pharmacological profile due to its primary affinity with µ-opioid receptors and lower affinity with κ and delta opioid receptors [146].

The addictive liability of oxycodone is determined by the activation of these receptors, which modulate neurotransmission, particularly dopamine pathways in the mesolimbic system, mostly linked to reward and reinforcement, thereby providing to the addictive liability of oxycodone [147].

It has been established that oxycodone and morphine elicit divergent neurobiological responses within the mesolimbic reward pathway, particularly involving the nucleus accumbens and ventral tegmental area. Within the mesolimbic trajectory, dopamine functions as the primary reward mediator, liberated upon the binding of opioid agonists to µ-opioid receptors. This binding event antagonises the inhibitory influence of gamma-aminobutyric acid (GABA) interneurons, which typically regulate dopaminergic efflux into the synaptic cleft. The attenuation of this inhibitory tone facilitates increased dopaminergic neurotransmission throughout the mesolimbic pathway. A critical kinetic distinction exists between the two ligands: morphine-induced dopamine elevation is characterised by transient inhibition of GABA, resulting in a brief dopaminergic pulse. In contrast, oxycodone elicits a more persistent suppression of GABAergic signalling, maintaining stable and protracted dopamine concentrations within the reward circuit [148].

The observed divergences in signalling kinetics and circuit activation may underpin the heightened dependence of liability and abuse potential of oxycodone relative to other prescription opioids. These neurobiological distinctions are often exploited through the mechanical degradation of ER formulations, specifically via crushing or mastication, to facilitate rapid delivery via oral ingestion or intranasal insufflation. Such methods bypass intended delivery mechanisms, leading to accelerated absorption and a more pronounced reinforcement effect [20,149].

#### 3.3.2. Oxycodone Overdose Management and Toxic Dose

Previous research showed that toxicological assessments indicate that counterfeit oxycodone formulations are frequently adulterated with lethal concentrations of fentanyl, serving as a primary driver of escalating opioid-related mortality. The distinct pharmacokinetic and pharmacodynamic profiles of fentanyl relative to oxycodone demand a re-evaluation of standard overdose protocols. Given these differences, effective reversal may require extended clinical observation or more intensive interventions, such as continuous naloxone infusions [150]. Moreover, the clinical utility of opioid immunoassays in emergency settings is hindered by divergent detection sensitivities and cross-reactivity. For example, several oxycodone assays demonstrate significant reactivity with its metabolite, oxymorphone, whereas synthetic analogues such as fentanyl frequently evade standard screens, necessitating specialised, targeted toxicological panels for definitive identification [151].

Consequently, the precise and rapid identification of the offending opioids—particularly in the context of polydrug intoxication—is critical for tailoring clinical interventions and improving prognostic outcomes [150]. The exceptional potency and rapid pharmacokinetics of fentanyl demand a therapeutic approach distinct from the protocols established for oxycodone intoxication. Clinical management must specifically account for the high risk of protracted respiratory compromise, which often necessitates escalated monitoring and the repeated—or continuous—administration of opioid antagonists to achieve sustained ventilatory stability [152].

In contrast, while the progression of respiratory depression in oxycodone toxicity is typically more insidious, the potential for severe central nervous system and ventilatory impairment remains acute. Clinical management frequently necessitates sustained respiratory intervention and diligent monitoring for recrudescent toxicity, which may occur as the antagonistic effects of naloxone diminish despite an initially successful reversal [23].

Additionally, the detection of ultra-potent synthetic analogues such as carfentanil within counterfeit formulations further exacerbates the complexity of overdose reversal. These agents can induce refractory respiratory depression that may prove resistant to conventional naloxone titration, highlighting a critical need for advanced therapeutic modalities, such as monoclonal antibody-based immunotherapy [152]. In light of the heterogeneous potency and chemical purity of illicitly manufactured opioids, effective overdose mitigation strategies must prioritise the communication of risks inherent in counterfeit formulations. Such efforts should facilitate the utilisation of harm reduction technologies—including drug-checking services and fentanyl test strips—to provide critical transparency regarding the presence of high-potency adulterants within the unregulated drug supply [153,154].

This multidimensional challenge highlights the critical imperative for comprehensive public health frameworks and pioneering clinical modalities to attenuate the morbidity and mortality associated with oxycodone-related and broader opioid-induced toxicities. Optimised management of opioid use disorder within an era defined by high-potency synthetic analogues necessitates an integrated therapeutic approach. This includes advanced pharmacotherapeutic strategies—specifically modified buprenorphine/naloxone induction protocols—synergised with evidence-based behavioural interventions such as cognitive behavioural therapy [155].

Such multidisciplinary strategies are fundamental to addressing the multifaceted neurobiological and behavioural correlates of opioid use disorder, a necessity further underscored by the heightened lethality inherent in the contemporary, unregulated toxicological landscape [155].

However, in these programmatic developments, a substantial deficiency remains regarding the long-term knowledge of retention and systematic evaluation of Overdose Education and Naloxone Distribution training among healthcare practitioners. This persistent gap underscores a critical imperative for more enduring pedagogical frameworks and iterative educational initiatives to ensure sustained clinical competency in emergency interventions [156].

To address the escalating opioid crisis effectively, the widespread dissemination of naloxone must be coupled with comprehensive educational initiatives regarding its administration. These programmes must place particular emphasis on the necessity for titrated, multi-dose protocols, required to counteract the profound respiratory depression associated with high-potency synthetic analogues [157].

Moreover, frontline responders frequently encounter presentations of opioid-induced cardiopulmonary arrest, particularly in the context of illicitly manufactured fentanyl. This clinical reality underscores the imperative for expedited and ubiquitous naloxone delivery to facilitate rapid reversal during the critical prehospital window [158].

The unprecedented surge in fatalities associated with illicitly manufactured fentanyl (IMF) underscores a critical imperative to optimise harm reduction frameworks and broaden access to substance use disorder therapies. This necessity is further compounded by the diagnostic challenges inherent in certain IMF-related deaths, where a paucity of pathognomonic evidence or traditional toxicological markers may confound definitive cause-of-death determinations [158].

#### 3.3.3. Oxycodone Withdrawal Symptoms and Treatment of Withdrawal Syndrome

Oxycodone is associated with a high propensity for inducing withdrawal syndrome due to its rapid onset and shorter half-life, leading to significant fluctuations in opioid levels and subsequent neurochemical imbalances [159]. The physiological manifestation of opioid withdrawal is primarily driven by a deficit in μ-opioid receptor occupancy, resulting from either a reduction in exogenously administered opioid agonists or diminished endogenous opioid signalling due to receptor downregulation [160]. This neuroadaptation subsequently results in a cascade of physiological responses upon cessation or reduction in oxycodone, manifesting as a complex constellation of withdrawal symptoms [161]. Specifically, oxycodone, a semisynthetic derivative of thebaine, has been a significant contributor to the opioid crisis due to its misuse and abuse potential. This inherent liability is underscored by historical concerns dating back to the 1940s regarding its abuse potential, which was considered comparable to that of heroin [23].

The over-prescription of oxycodone in North America has exacerbated this issue, propelling it to epidemic proportions and making it the primary opioid implicated in the early stages of the opioid use disorder crisis. However, increased restrictions on its prescription and distribution have led to its displacement by fentanyl and its analogues in the illicit drug market [26]. Despite sharing structural and pharmacological similarities with morphine, both primarily exerting their effects through the µ-opioid receptor, oxycodone exhibits distinct neurobiological and pharmacological properties that contribute to its unique abuse liability [23]. The rapid development of tolerance and the severity of opioid withdrawal, characterised clinically by symptoms such as gastrointestinal distress, sweating, rhinorrhoea, lacrimation, yawning, restlessness, and mydriasis, further contribute to its potential to produce physical dependence [162,163].

Opioid withdrawal from oxycodone is managed using the same principles as withdrawal from other short-acting opioids. Research emphasises that good withdrawal control is critical, both to relieve suffering and to prevent relapse or overdose during this high-risk period [125,164,165,166]. Previous research shows that the first line for opioid withdrawal syndrome is opioid agonists or partial agonists, such as methadone and buprenorphine. These substances reduce symptoms, cut cravings, and improve retention in treatment. It was reported in the literature that maintenance with these drugs is superior to detoxification alone [164,165,167].

#### 3.3.4. Oxycodone-Related Deaths

A fatal overdose of oxycodone is usually caused by respiratory depression, but there is no single definitive ‘lethal dose’, as the risk is significantly influenced by tolerance, alcohol and benzodiazepines, the route of administration and any underlying medical conditions [106,168,169].

Behaviour prior to death most commonly reflects increasing opioid sedation: lethargy, a decline in consciousness, shallow breathing and progressive hypoxia, rather than sudden agitation [170,171]. The typical clinical picture prior to death includes constricted pupils, bradypnea, hypotension, bradycardia, hypothermia and impaired consciousness [172]. In fatal cases described following oxycodone use, deep sleep and loud snoring were also observed before the bodies were found, which is consistent with progressive airway obstruction and respiratory failure [171]. The narrow time window is critical because, once hypoxia sets in, brain damage can occur within 3–6 min, followed by bradycardia, arrhythmias and cardiac arrest [170].

Research does not support the existence of a single universal lethal dose of oxycodone. In an analysis of adverse events in cancer patients, the median dose of oral oxycodone at the first report of respiratory depression corresponded to approximately 30 mg/day in terms of oral morphine, and half of the cases occurred within 30 days of starting treatment. This shows that severe toxicity can occur even at relatively low clinical doses in susceptible individuals [173].

In studies involving volunteers, 20 mg of oral oxycodone reduced ventilation by 28%, whilst combining it with alcohol exacerbated this effect by a further 19% and increased the number of apnoea episodes; older people showed a stronger response [174].

In another study, 20 and 40 mg of oral oxycodone were sufficient to induce significant respiratory depression, although in healthy young adults this was still within the experimentally controlled range [175]. Post-mortem blood concentrations are more useful than the reported dose, but they also have significant limitations. In large series of fatal poisonings, the median or mean concentrations were generally higher in fatal cases than in other deaths: 0.30 µg/g versus 0.05 µg/g in a Swedish series of 1236 autopsies, an average of 0.40 mg/L in 70 cases from New South Wales, and a median of 0.43 mg/L in 24 deaths attributed solely to oxycodone [169,176,177]. At the same time, the ranges were very wide, from 0.06 to 53.0 mg/L in the Australian series, and more than half of the ‘oxycodone alone’ deaths in the older series had concentrations of ≤0.5 mg/L [176,177]. The interpretative thresholds are therefore only indicative. Data from femoral blood analyses suggest that concentrations > 0.2 μg/g are likely to contribute to toxicity, but levels of up to 0.3 μg/g may be found in individuals who tolerate opioids without fatal consequences [178]. A meta-analysis from 2026 reports that the mean oxycodone concentration in peripheral blood samples, obtained from lethal overdose cases, equalled 0.37 mg/L. As such, the mean concentration differed between mono- and mixed-drug intoxication, amounting respectively to 0.52 mg/L and 0.29 mg/L [179].

The most common cause of death in contemporary cases is not ‘pure’ oxycodone, but fatal poly-drug intoxication. In a series of 172 deaths in Palm Beach, only 18 were attributed to oxycodone toxicity, whilst 117 were attributed to mixed toxicity [180]. In the Australian series of 70 cases, other psychoactive substances were detected in all cases, most commonly hypnotics, other opioids, antidepressants and alcohol [176]. According to Swedish data, people who died from oxycodone poisoning were 5.6 times more likely to have other CNS depressants in their blood, and such drugs were found in 81% of cases [169]. This is consistent with the wider literature on opioids: combining them with alcohol and benzodiazepines increases their toxicity and can lead to death at lower opioid concentrations than with single-substance exposure [181,182].

The risk also increases when the form of medicine is altered. OxyContin tablets, available in doses of 10–160 mg per tablet, are designed to be released slowly over approximately 12 h, but crushing or dissolving them causes the dose to be released rapidly, leading to uncontrolled increases in blood levels [171,172]. In the NSW series, 21.4% of the deceased had injected the tablets immediately before their death, and 30% did not have a prescription for oxycodone [176].

The forensic interpretation of death following oxycodone ingestion is difficult because tolerance, post-mortem redistribution and co-intoxication can obscure the relationship between post-mortem concentration and toxic effect [171,178]. Post-mortem redistribution may be significant: in the two fatal cases described, the concentration in peripheral blood 10 days after death was more than 10 times higher than in a sample collected 72 h after death [171]. Therefore, toxicological findings should not be interpreted in isolation but in conjunction with the circumstances of death, post-mortem examination, the type of site and the biological sample, and the time of collection [171,183].

Metabolic markers may provide additional information when interpreting oxycodone-related deaths. Metabolite-to-parent drug ratios can help characterise the timing and extent of oxycodone metabolism. A low noroxycodone/oxycodone ratio has been proposed as a sensitive indicator of acute oxycodone intake. On the other hand, the oxymorphone/oxycodone ratio is influenced by CYP2D6 activity and should therefore be interpreted together with the CYP2D6 genotype or phenotype rather than used as an independent marker of intoxication [184,185].

Beyond targeted metabolite measurements, untargeted post-mortem metabolomics represents a promising complementary approach. Using untargeted mass spectrometry-based metabolomics, Elmsjö et al. identified alterations in several acylcarnitines in post-mortem femoral blood and proposed these metabolites as candidate biomarkers for fatal oxycodone-related intoxication [168]. Such an approach may capture endogenous metabolic disturbances associated with fatal intoxication that are not reflected by oxycodone concentrations alone. Importantly, these findings remain exploratory and require validation in independent cohorts before metabolomic signatures can be applied routinely in forensic casework.

The combination of oxycodone and metabolite concentrations, metabolite-to-parent ratios, pharmacogenetic information, and emerging metabolomic biomarkers may provide a more informative framework for interpreting oxycodone-related deaths than any single toxicological parameter alone.

### 3.4. Methods of Oxycodone Detection

The increasing use of oxycodone, both for medical and non-medical purposes, necessitates the selection of suitable materials and the most sensitive methods for detecting this chemical compound, as well as its metabolites, particularly noroxycodone and oxymorphone [186]. Noroxycodone, compared to oxymorphone, is found in post-mortem blood at higher concentrations, with a median of 0.051 mg/L [179]. The aim is not only to initiate rapid, targeted treatment in cases of poisoning and to monitor the condition of hospitalised patients experiencing severe pain, but also to determine the cause of death in cases of suspected overdose [186].

Choosing the appropriate method can present challenges. Consideration must be given to the sample to be collected for analysis—its stability and clinical utility in relation to the patient’s condition—the patient’s consent, past medical history, current and recent medication history, and the estimated time elapsed since the substance was ingested. The possibility of polysubstance intoxication must be taken into account. In the case of post-mortem patients, particular attention must be paid to the time elapsed since death. Furthermore, environmental contamination may occur. Different methods vary in terms of their sensitivity and specificity, making them more suitable for either screening or confirmatory testing.

Below we try to briefly present the methods used in oxycodone toxicology and compare the methods (chromatography and immunoassay) and materials (blood, urine, vitreous humour, oral fluid, hair) most commonly used in oxycodone toxicology.

The current gold standard for diagnosing oxycodone poisoning is the measurement of blood concentrations using chromatography methods, especially liquid chromatography–mass spectrometry (LC/MS) [186] and liquid chromatography–tandem mass spectrometry (LC-MS/MS). This method is replacing the previously used gas chromatography–mass spectrometry (GC/MS). GC/MS is less sensitive than LC/MS. It requires the conversion of opioids into their more volatile derivatives, as well as deglucuronidation. As the test material was converted into gas, the active substance had to be thermolabile and not decompose during the transition from a liquid to a gaseous state. These drawbacks have been overcome, as chemical compounds can now be detected at even lower concentrations [187].

LC/MS is not restricted by this limitation [187,188]. This method is characterised by high sensitivity and specificity. It enables the detection of chemical substances even in trace quantities [186]. With appropriate extraction using LC-MS/MS, oxycodone can be detected in the blood at a concentration of 0.1 ng/mL [189]. Furthermore, it is highly selective and can be used to distinguish oxycodone from other opioids, as well as from its metabolites, whilst also enabling quantitative analysis [190], even if other compounds are present in the biological samples [191]. Another advantage of using LC/MS is the ability to analyse a wide variety of samples. Not only are blood and urine suitable for testing, but with appropriate sample preparation, results can also be obtained from saliva, hair, or tissue samples [192].

However, the drawbacks include the cost of the tests, the need for specialist equipment and trained staff, and the time-consuming nature of the method [186].

New chromatographic methods increase the sensitivity of analyses, enabling substances to be distinguished by accurate mass and elemental composition. This is achieved by adding additional mass spectrometry instruments or by coupling chromatography with instruments of exceptional sensitivity, such as a quadrupole coupled with a time-of-flight analyser. New methods now include, for example, high-resolution mass spectroscopy (HRMS) [193] and Ultra-High-Performance Liquid Chromatography–tandem mass spectrometry (UHPLC-MS/MS), which can detect oxycodone at a concentration of 0.02 ng/mL [194].

Whilst chromatographic methods are ideal for verifying results and the specific identification of chemical compounds, they are not used in screening due to the long time required to obtain results. For screening, immunoassay methods are more preferred [195], for example, enzyme-linked immunosorbent assay (ELISA) [196], enzyme-multiplied immunoassay technique (EMIT), cloned enzyme donor immunoassay or kinetic interactions of microparticles in solution (KIMS) [197].

Immunoenzymatic methods are based on the specific binding of a labelled antibody to an antigen present in a biological fluid [198]. In the case of ELISA tests, depending on how the coloured product (which indicates the presence of the compound or group of compounds being tested) is obtained, we can distinguish between different types of tests: direct, indirect, sandwich and competitive tests [199].

In direct methods, the labelled antibody binds directly to the antigen attached to the plate. In indirect methods, the labelled antibody binds to the antigen via an additional antibody. The sandwich ELISA method involves binding one antibody to the plate, and then, after the antigen present in the test material has bound to this antibody, adding another labelled antibody. If two types of antibodies are used in the method, labelled antibodies and others act as reaction inhibitors (which bind to the plate in the absence of antigen, thereby preventing a colour change); this is referred to as a competitive ELISA [200,201].

Immunoassay methods can produce four types of results: true positives and true negatives, as well as false positives and false negatives [200]. False-negative results are more problematic from a diagnostic perspective than false-positive results. Positive results require confirmation using more specific methods, such as chromatographic methods. Such confirmation is not usually carried out in the case of negative results, which may result in some cases being missed [202].

The results obtained depend on the type of immunoenzymatic assay used, its specificity, and detection capacity. Results may vary depending on the manufacturer of the test [203]. There are tests that specifically detect oxycodone [203,204], but there are also those that detect an entire group, such as opioids, allowing only the identification of the group rather than a specific compound [203,205]. Tests designed to detect a whole group of opioids may sometimes produce false-negative results if the sample contains oxycodone [205]. This is why specific tests dedicated to oxycodone should be used when poisoning with this substance is suspected. This would prevent false-negative results from occurring [197]. There are oxycodone-directed ELISA kits that detect this compound at a concentration of 1 ng/mL in oral fluid [206].

A number of compounds can interfere with the test results. False-positive results may be caused, among other things, by the presence of other similar chemical compounds in the sample, including naloxone [187,207], as well as the metabolites produced after consuming poppy seeds [204,205]. Another factor affecting the result is antibiotic treatment with quinolones if undertaken shortly before the test [204,208]. So far, it has not been possible to determine conclusively what causes this situation [208].

Depending on the test used, metabolites may not be detected [187]. In addition, the test may produce a negative result if the biological sample is too diluted or if too much time has elapsed since the sample was collected [209].

The advantages of immunoenzymatic methods include the speed with which results are obtained, the automated nature of the method and its simplicity, meaning that specially trained staff are not required [204]. A comparison of the LC/MS method with immunoassay methods is presented in Table 2.

The appropriate method should be chosen at the stage of selecting the appropriate diagnostic material, considering the patient’s condition, the availability of the material, and the time at which the oxycodone entered the patient’s system [210].

One of the basic diagnostic materials is blood [211]. It is suitable for collection from both living and deceased patients. The difference lies in the site from which the material is collected [210].

In ante-mortem patients, 5 to 10 mL of blood is collected from the antecubital vein into a tube containing anticoagulants [210] and preservatives, such as sodium oxalate or sodium fluoride [212]. In post-mortem patients, blood is obtained from the heart and from peripheral blood, particularly from the femoral vein. In post-mortem blood from the heart chambers, xenobiotics are present higher concentrations, making it possible to detect them even though only trace amounts are present in peripheral blood [210]. In the case of oxycodone, however, this relationship has not been not observed, and the ratio of the drug’s concentration in peripheral venous blood to that in cardiac ventricular blood is approximately 1 [213].

When conducting post-mortem blood tests on patients, the phenomenon of post-mortem redistribution must be taken into account [214,215]. This is due to a change in tissue integrity caused by the breakdown of cells during autolysis. This results in the facilitated diffusion of chemical compounds between the blood and the surrounding tissues, primarily the muscles [216]. During life, the liver and the digestive tract act as storage organs, and after death, xenobiotics are released from them [217]. Furthermore, after death, blood flows through the blood vessels passively, partly as a result of gravity [218], and also as a result of changes in pressure, initially due to post-mortem rigour and subsequently due to the gases released during decomposition [215]. As a result of these processes, post-mortem blood concentrations do not always accurately reflect concentrations during life. This can distort the picture and lead to erroneous results [215,216].

Blood from the femoral vein following death exhibits only small fluctuations in concentration compared to that from the rest of the body and is the most resistant to the effects of post-mortem redistribution. For this reason, it is the best material for quantitative analysis of xenobiotics in post-mortem patients [217].

Burns and injuries can also make it difficult to interpret blood test results [214], as these factors can affect the body’s water and blood content [219].

Blood and urine samples should be stored in the fridge or freezer to slow down the decomposition of the material and the xenobiotic contained in it [220]. In 2009, Al-Asmari et al. found that oxycodone concentrations remained stable in stored blood and urine for one month at a temperature of 4 °C, and for six months at a temperature of −20 °C [221].

New techniques are improving the usability of blood. One such method involves the use of dried blood spots (DBSs). Special porous cellulose paper makes it possible to collect the sample. Unlike traditional blood collection, this process is easier and less stressful for the patient, as smaller volumes are collected, and the procedure is less invasive [222]. In infants, samples may be taken from the heel vein to assess exposure to the active substance during pregnancy [223].

This method is simple and inexpensive. The entire sample collection process is automated. The collected samples can be easily stored and transported. The substances they contain are less likely to degrade. On the other hand, the results obtained using this method are not equivalent to those obtained from whole blood. Due to the small amount of sample collected, it is not possible to repeat the tests using previously collected samples if necessary [224]. Although the samples are ideal for investigating acute conditions, they are not suitable for use in emergencies, as the sample must be left to dry for at least 2 h. However, DBSs are ideally suited for transport to a laboratory situated a two-hour drive from the site where the sample was collected, as the sample will have time to dry during transit, and the whole procedure will be simpler and sample will take up less space [225].

DBSs are used for detection of oxycodone. The results obtained are similar to those achieved when using liquid blood. The sample can be stored for a month in a dry place without any loss in xenobiotic content. However, the entire procedure is less complicated than when using liquid blood. There is no need to insert a needle into the patient’s vein [224].

For patients who are alive and dependent on psychoactive substances, urine tests are the gold standard [203]. These tests should be carried out without prior notification to the patient [226].

The advantages of urine as a diagnostic sample include the ease and low cost of collection, as well as the non-invasive nature of the method [227].

Urine is not a flawless sample. The detection window for the chemicals it contains is short [228]. In the case of oxycodone, it is up to 4 days [229]. If urine is collected from patients post-mortem, death must have occurred at least one hour after ingestion of the compound to allow the chemical to have time to pass into the urine [210].

Based on tests carried out on 15 urine samples collected from a single patient following the ingestion of IR oxycodone, in 2020, Truver et al. [230] demonstrated that extending the detection of oxycodone in urine to include its metabolites, such as noroxycodone or glucuronide compounds, allows the detection window to be extended by 48 h. A limitation of this study was that, as it was conducted on only one individual, it does not account for individual differences and may not be representative of the wider population.

In certain specific situations, collecting a urine sample also involves an intrusion into the patient’s privacy [231]. Without special supervision, this makes it possible to swap the urine sample for urine from someone who does not use illegal substances or for artificial urine, or to add a substance to the urine sample that falsifies the results, which may occur, for example, in the case of people with substance abuse problems or those with a criminal record [232].

In post-mortem toxicological examinations, the vitreous humour obtained from patients’ eyes also provides valuable diagnostic material [210].

The vitreous body is located at the back of the eye, behind the lens and the ciliary body, and is surrounded by the retina, the choroid and the sclera. Despite its high water content, ranging from 98 to 99.7 per cent, the vitreous body has a gel-like consistency due to the presence of fibrillar proteins, mainly collagen. It has a volume of approximately 4 mL in each eyeball [233]. Xenobiotics enter the eyeball by passive diffusion across the blood–vitreous barrier. This barrier is in fact composed of two separate barriers: an inner barrier formed by the endothelial cells of the blood vessels and an outer barrier derived from the retinal pigment epithelium [233,234].

In 2009, Knittel et al. [234] demonstrated, based on 57 samples collected from patients during autopsies, that the vitreous humour is suitable for confirming the presence of oxycodone in the body. At the same time, they noted that it is not adequate material for determining blood concentrations.

The advantages of the vitreous humour as a diagnostic material include the ease with which it can be collected and the stability of its environment regarding xenobiotics. Thanks to its location and the presence of the blood–vitreous barrier, this material is protected from the harmful effects of external factors, trauma and microorganisms, as well as from decomposition [233,234].

Bévalot et al. [233] note that storing a sample of vitreous humour for one year at −20 °C does not affect the oxycodone content of that sample.

Oral fluid is one of the sample materials that has become increasingly popular in toxicological studies involving patients with substance use disorders [235]. This is due to the fact that the procedure does not intrude on the patient’s privacy to the same degree as a urine sample [236]. Thus, it is an ideal material for screening purposes, e.g., for drivers or employees. The method is also relatively inexpensive and allows for simple, non-invasive sample collection for testing [237].

Oral fluid is not the same as saliva, as saliva is merely a secretion of the salivary glands, and, in addition to gland-secreted substances, oral fluid contains other substances [235]. Oral fluid consists of water (99%), electrolytes, proteins and enzymes, as well as oral microbiota, epithelial cells, and fragments of ingested food [238].

It is thought that oral fluid provides a more accurate reflection of blood concentrations than urine [239], except in cases where the substance was taken orally or smoked [235]. Studies conducted to date on the correlation between oral fluid concentrations of oxycodone and plasma concentrations do not support this theory [237,240].

In 2011, Hardy et al. [240], based on 139 paired samples collected from the oral cavity and the blood of 43 patients treated with oxycodone SR at an average dose of 40 mg per day, demonstrated that the correlation coefficient was less than 0.5. Concentrations in the oral cavity were also significantly higher—averages of 336 μg/L for oral fluid and 38.2 μg/L for blood were detected.

A study conducted by Heiskanen et al. in 2014 [237] revealed an even lower correlation between these two biological fluids, estimating it at 0.267, with concentrations in oral fluid on average almost 15 times higher than in plasma. The cause of this phenomenon is believed to be most likely the presence of the pyrilamine transporter, which was first detected in the blood–brain barrier of rats [241]. This transporter is an energy-dependent antiport that transports hydrogen ions and organic cations in opposite directions across the membrane [242]. Oxycodone is one of the substances following this path [241]. However, the exact mechanism remains unknown.

Another advantage of using oral fluid as a diagnostic sample is its ability to detect substances that have been recently ingested, thanks to the rapid transfer of the active substance into the fluid [239].

In the case of oxycodone, a study conducted by Cone et al. in 2015 [243] on 12 patients, after collecting 300 oral fluid samples and 276 blood samples using LC/MS, showed that following the ingestion of 20 mg of CR oxycodone, it is possible to detect oxycodone as early as 15 min after oral administration. Meanwhile, in the blood, following ingestion of the same dose, the active substance is detectable between 0.5 and 2 h after administration. An additional advantage demonstrated by Cone’s team was that the detection of oxycodone and its metabolites, mainly noroxycodone, in oral fluid persisted on average twice as long as in blood.

The disadvantages of oral fluid as a diagnostic sample include its small volume (not exceeding 1 mL) and its variability due to various factors such as age, gender, diet, and health status [244].

Oral fluid, like blood, should be stored at fridge or freezer temperatures. However, compared with blood, oral fluid is more stable and exhibits less variation in the concentration of the tested substance [245]. At −70 °C, these samples can be stored for up to 22 months [237].

Human hair is a material that, in turn, has an exceptionally wide detection window. Xenobiotics can be detected in it for up to 90 days [246,247], depending on the rate of hair growth [227]. This remarkable property is due to the presence of keratin, which is capable of retaining xenobiotics within the hair structure for months or even years [248].

Xenobiotics can enter the hair via three routes. The first is diffusion from the blood supplying the hair during the growth phase. The second is the absorption by the hair shaft of substances secreted in sweat and sebum. The third route is deposition on hair of substances originating from the environment. The detection of xenobiotic metabolites makes it possible to distinguish whether a substance has been incorporated into the hair structure following use of the substance or following environmental exposure [249].

Stowe et al. [250] used LC-MS-MS to analyse hair samples from American workers who reported using oxycodone. The hair samples, totalling 208 samples, were collected between 2014 and 2024. Oxycodone was detectable in every one of these samples, and the greater the reported dose of the drug consumed by the subject, the higher the concentration.

Another advantage of hair as a diagnostic material is that it allows us to determine whether exposure to the substance was a one-off occurrence or whether it was repeated, and at what intervals [248].

A drawback of this material is that it cannot be used to detect xenobiotics if the substance was ingested shortly before death [247]. Sometimes it can take as long as 10 days for a substance incorporated into the hair structure to reach the surface of the skin [249].

Hair samples should be collected prior to post-mortem examination, from areas that are clean and free from visible damage, as blood may interfere with the test results [251]. The best place to take a hair sample is at the back of the head, near the sagittal suture, in an area known as the vertex, as this is where hair length varies the least and growth is fastest. If necessary, hair can be sampled from other areas. Once the hair has been cut in a contamination-free environment using specially designed, sterilised instruments, it can be stored indefinitely, as it does not undergo significant decomposition over time. It is important to clearly mark the part of the hair that was closest to the body, as during the analysis the hair is cut into segments and the concentration of the active substance in those specific segments is assessed in order to determine when the person came into contact with the substance [249].

As hair is exposed to harmful external factors such as water, light and temperature, and is sometimes subjected to cosmetic treatments that alter its structure, it is not always suitable for diagnostic purposes. The material within it may be damaged. In such cases, nails are a similar material with comparable clinical utility [252].

Nails are very similar to hair. They are made of the same substance, keratin, and xenobiotics enter them via the bloodstream, sweat, sebum or saliva; they may also be absorbed from the external environment [253]. They have a long detection window, as long as 5–14 months [254], which allows substances, such as oxycodone, that had been ingested months or even years before the test to be detected. Nails are characterised by steady, consistent growth, ranging from 1.9 to 4.4 mm per month on the hands, and approximately 30–50% more slowly on the feet [255]. Sample collection is simple and non-invasive. Samples can be stored at room temperature without any risk of damage to the material or compromise to the stability of the xenobiotic. Furthermore, nails do not contain melanin, which could interfere with the result [253].

In 2015, Shu et al. [255] demonstrated that the concentration of xenobiotics, such as oxycodone, is higher in fingernails than in toenails, and also that the parent compound (oxycodone) is incorporated to a greater extent than its metabolites (e.g., oxymorphone). A comparison of various types of samples is presented in Table 3.

### 3.5. Limitations

To our knowledge, in toxicology, bones and bone marrow are useful diagnostic materials when no other materials are available. We intended to include these materials, but due to research data, databases and language limitations (due to a language barrier we were able to use only articles written in English or Polish), in the end, they had to be excluded from our study, as we have so far been unable to find any study validating the usefulness of bones as a diagnostic material in cases of oxycodone poisoning by using keywords such as “bone”, “bone marrow”, “oxycodone” or “poisoning”. However, this could be a perfect topic for future discussions.

Furthermore, our aim was not to validate the methods used in the toxicological detection of oxycodone. For this reason, we did not focus on describing specific methods and did not include them in our paper, nor did we address their formal validation or methods of sample preparation for specific tests. We have merely presented the types of materials for which there is a scientific basis for the detection of oxycodone, along with the most commonly used methods in toxicology.

## 4. Discussion

Oxycodone has played a large part in the evolution and expansion of the phenomenon known as the opioid crisis [26]. The first concerns about the addictive potential of oxycodone appeared soon after its release [23]. In terms of misuse, oxycodone has a few properties which make it both more likely to be prescribed and abused over morphine, including higher bioavailability in the oral route of administration [256] and faster onset of action [26]. Huddart et al. suggest that due to the lower tendency of oxycodone’s toxic metabolites to accumulate in tissue, it has a slightly lower tendency to cause side effects [39]. However, a study from 2010 has revealed that oxycodone’s rapid onset of action corresponds not only with its analgesic effect, but also with respiratory compromise, which is more severe for oxycodone compared to equivalent doses of morphine [257]. Alternatively, the diversity of the individual characteristics of patients has an impact on the pharmacokinetic and pharmacodynamical parameters of oxycodone. Patients with renal or hepatic dysfunctions, as well as elderly patients, require particular supervision during oxycodone therapy, as—due to a drug clearance impairment—they may be more liable to potential adverse effects and complications [39]. On the other hand, in children, the clearance of oxycodone can be up to 40% higher than in adult patients. The metabolism linked to CYP enzymes, especially isoenzymes CYP3A4 and CYP2D6, makes oxycodone prone to interactions with other medications, especially when orally administered [36]. It is believed that there is no association between food intake and the pharmacokinetic or pharmacodynamic properties of CR oxycodone [39,40,258,259]. Nevertheless, a considerable change in the bioavailability and maximum blood concentration of IR oxycodone has been reported following high-fat meal consumption [258].

The type of opioid receptors that oxycodone interferes with remains unconfirmed. Both oxycodone and morphine are considered as full [260] agonists of the Mu opioid receptor [261]. However, in a study in 2023, Marie et al. classified oxycodone and its metabolite oxymorphone in a group with partial Mu agonists [26], while some sources indicate that oxycodone is majorly a selective MOR agonist [81] and has a strong affinity for μ opioid receptors. Others imply that oxycodone interferes with kappa and delta opioid receptors as well [26], but with much lower affinity than for mu receptors. It binds DOR more potently than KOR. Oxymorphone, oxycodone’s metabolite, has higher affinity for KOR [26]. It is also worth noting, that—based on rat MOR studies—it has been revealed that oxymorphone’s affinity for the mu receptor is 100 times higher than that of oxycodone [20]. Opioid antinociceptives are usually targeted at mu opioid receptors [260]. MOR involvement in the analgesic effects of oxycodone remains undisputed [262]. However, in 1997, a role of KOR in the antinociceptive effect of oxycodone was suggested [263]. Other studies have reported that some of oxycodone’s analgesic properties are attributed to kappa receptor activation [263,264]. A review from 2008 classified oxycodone as a kappa agonist [21]. This may indicate why oxycodone might be a better choice for some kinds of pain, as, for example, during inflammation, the number of kappa receptors within the nervous system is elevated [265]. Furthermore, in another review article, the authors reported that oxycodone has a higher affinity for KOR than MOR and mentioned oxycodone’s impact on DOR [22]. In terms of the delta opioid receptor and its role in oxycodone’s antinociceptive utility, according to a study from 2016, it has not been fully clarified whether it takes part [262]. In addition, other authors have demonstrated in their research that DOR antagonists can effectively counteract the analgesic properties of oxycodone in mice [262]. Another study from 2019 acknowledged oxycodone’s impact on the delta receptor in mice, as naltrindole, which is a selective DOR antagonist, mitigated a few of oxycodone’s side effects [266].

Oxycodone’s affinity for opioid receptors ranges between 1/10 and 1/40 compared to morphine [22]. Even though oxycodone exhibits the highest affinity for the mu opioid receptor, it is between 5 and even 40 times lower in comparison with that of morphine [26]. Regarding only the mu opioid receptor, Marie et al. have reported that oxycodone activates MOR with lower potency than morphine [26]. In fact, there exists a phenomenon of an affinity/potency paradox, as the analgesic potency of oral oxycodone is equal to that of oral morphine or even higher, while its ratio is estimated to be between 1:1 and 2.2:1 [265]. Oxycodone has an oral advantage over oral morphine due to its higher bioavailability, as these substances achieve a bioavailability of 60% and 33% respectively [267]. Based on human studies, oral oxycodone has a 1.5-times higher antinociceptive potency than morphine [268]. Other sources estimate that this value may be two times higher [267]. Other than its antinociceptive effects, the potency of oxycodone is triple the potency of morphine [269]. This means that lower doses of oxycodone can cause adverse effects equivalent to those of morphine [265], including dizziness or somnolence [269].

When oxycodone was put on the market as OxyContin in 1996, Purdue Pharma tried to decrease the risk of addiction by formulating sustained-release pills. However, just by crushing the pill, the user can easily apply the substance to themself by snorting or injection [23,27]. Oxycodone has a strong affinity for μ opioid receptors, which are responsible for euphoria and physical addiction [21]. Soyka et al. [147] credit these effects to its ability to modulate dopamine pathways in the mesolimbic system, responsible for reward. In contrast to morphine, use of oxycodone is associated with a heavier „wanting” and more likely triggers compulsive behaviour [20]. By more persistent GABA suppression, oxycodone facilitates maintaining stable levels of dopamine within the circuit of reward [148]. What increases the risk of dangerous implications of counterfeit oxycodone formulation use is the fact they might be polluted with lethal concentrations of fentanyl, so in oxycodone overdose cases, the abuser might need extensive medical intervention and targeted panels for identification [150,151]. If oxycodone gets contaminated with carfentanil, then the following respiratory depression might even be resilient to naloxone [152].

These days, the gold standard methods for oxycodone toxicity identification are liquid chromatography–mass spectrometry (LC/MS) [186] and liquid chromatography–tandem mass spectrometry (LC-MS/MS) [187]. Contrary to GC/MS, this technique allows for the detection of particular substances in lower concentration levels [187]. Moreover, LC/MS enables us to distinguish oxycodone from its metabolites or other opioids [190], and it can be used to test diverse samples, including not only blood and urine, but also hair, saliva and tissue extracts [192]. On the other hand, the biggest disadvantages of this method are the high costs and dedicated device demand; it is also very time-consuming [186]. However, for screening, the most common methods are immunoassay techniques [195]. Some of the cons of such tests are discrepancies between obtained results from tests produced by different companies [203]. The results are prone to be disturbed by the presence of other substances in the sample, for example, naloxone [187,207] and metabolites of Papaver seeds [204,205]; the concentration of the substance; and the time after the collection of the sample [209]. To investigate oxycodone in the sample, there is a variety of materials that can be used, including blood [211], urine [203], liquid obtained from oral cavity [240], hair [250], and post-mortem vitreous humour [234]. Urine tests remain the gold standard in detecting oxycodone in alive humans [203]. Investigating urine is on the one hand cheap and easy to conduct [227], but on the other hand limits the ability to detect oxycodone in the sample to up to 4 days only [229]. However, Truver et al. [230] have proved that metabolites of oxycodone can be detected within the following 48 h as well. The available literature exhibits distinct values of the limit of quantification (LOQ). Cone et al., using LC-MS/MS, in their study reported an LOQ of 50 ng/mL [270]. The method of UHPLC-MS/MS has enabled Pantano and colleagues to detect oxycodone at a concentration of 0.15 ng/mL only; however, the LOQ has been estimated at 0.5 ng/mL [271]. Moreover, in a 2016 study, the authors managed to detect oxycodone at a concentration of 0.05 μmol/L using a CoFe_2_O_4_-modified carbon paste electrode [272]. Knittel et al. [234] stated that the vitreous humour is a sufficient sample for post-mortem detection of oxycodone, but it should not be used to assess its concentration in the blood. Liquid obtained from the oral cavity better corresponds with oxycodone blood levels than urine [239], provided that oxycodone had not been consumed orally or snorted [235], though this theory has not been confirmed yet [237,240]. This type of sample is useful for quick detection of recently consumed oxycodone [239]. Hair samples facilitate detection of the substance even 90 days after it was consumed [246,247]. Nevertheless, as it takes the substance around 10 days to rise above its level in the skin [249], it is impossible to detect a xenobiotic taken soon before demise [247].

When conducting post-mortem blood research, due to post-mortem redistribution [214,215], the results might diverge from the actual concentration of oxycodone before fatality [210,211]. Other factors that might have an impact on the detection of oxycodone post-mortem in various samples are—among others—the route of administration and administered dose, as well as metabolic diversity, which includes different CYP genotypes [179]. Kriikku et al. have indicated that pharmacokinetic interactions of CYP3A4 inhibitors may increase the toxicity of oxycodone [273]. This has been confirmed by Jakobsson et al. in their study, as poor and intermediate CYP2D6 metabolisers were found to be involved in intoxication more frequently. In this group, the median oxycodone femoral blood concentration has been estimated to be 0.15 µg/g (1.05 mg/L), compared to 0.072 µg/g in extensive and ultra-rapid metabolisers (concentrations were detected using the LC-MS/MS method) [185]. The range of oxycodone concentrations in post-mortem femoral blood samples differs between studies, as does the lowest limit of quantitation (LLOQ) value. Using UPLC–MS/MS allows us to determine the lowest LLOQ, which ranges between 0.005 and 0.0095 mg/L among studies, compared to the range of 0.02 to 0.05 mg/L when GC-MS and GC-NPD methods are used [179]. There are some discrepancies in the values of post-mortem oxycodone concentrations in peripheral blood from intoxication cases. In research from 2020, it was estimated to be 0.005 to 13 μg/g (0.0053 to 13.78 mg/L—approximate calculation) [184]. A study from 2012 on 155 samples has assessed the range of post-mortem oxycodone blood concentrations to be between 0.02 and 3.70 mg/L, with a mean value of 0.40 mg/L. The mean concentration linked to fatalities caused primarily by oxycodone intoxication differed from those linked to incidental causes, amounting to 0.48 mg/L and 0.16 mg/L respectively [274]. In an article from 2022, the oxycodone concentration in femoral blood from intoxication cases ranged between 0.005 and 83 µg/g, with a median of 0.30 µg/g (0.32 mg/L—approximate calculation) [169]. This study supports the above-mentioned article from 2021 [185], as drugs which interfere pharmacodynamically with oxycodone have been found more frequently in samples from oxycodone-related fatalities [169]. The most recent systematic review from 2026 exhaustively summed up available data, estimating peripheral blood oxycodone levels in fatal intoxications in the range of 0.24–0.58 mg/L, with a geometric mean value of 0.37 mg/L. This study has indicated that oxycodone reaches higher blood levels in mono-intoxications, with a GM value of 0.52 mg/L (range 0.22–1.21 mg/L), than in mixed-drug intoxication fatalities, where the GM is 0.29 mg/L (range 13–0.65 mg/L). As such, the authors also support the hypothesis that drug interactions may contribute to the fact that a lesser level of oxycodone is capable of causing fatal poisoning. This is why only taking into consideration the blood oxycodone concentration is not a sufficient method to state whether this substance has directly caused a demise [179]. Jakobsson et al. imply that the blood oxycodone level alone is not enough to confirm lethal intoxication, as concentrations of >0.2 μg/g might contribute to poisoning, but in other individuals, a concentration of 0.3 μg/g may be very well tolerated [184]. Another example is that the therapeutic plasma levels of oxycodone may range between 0.005 and 0.10 mg/L and thereby correspond with concentrations of peripheral blood oxycodone detected post-mortem. Moreover, tolerant patients may have higher plasma concentrations of oxycodone. For example, in surgery and cancer patients, these concentrations may be up to 0.3 mg/L and even up to 1.1 mg/L, respectively [273]. The obtained data suggests that there is no special cut-off value for blood concentration only that can be used to evaluate if oxycodone accounted for a fatality [184], and this needs to be evaluated in further research. To gather as much credible data as possible, early detection (<72 h) [179], classification as single- or multidrug abuse [273], case history [179] and post-mortem redistribution [214,215] should be circumstantially analysed.

## 5. Conclusions

Amid a rising wave of fentanyl-related poisonings and deaths, the problem of inappropriate oxycodone use is often overlooked. However, it is a significant issue and should not be ignored in the discussion about the opioid crisis. Various countries, such as the USA, Australia, Sweden and Switzerland, are reporting an increase in the number of deaths caused by oxycodone use. At the same time, its good absorption, high bioavailability and efficacy in relieving severe pain make it a popular choice in medical practice. It has found applications in a wide range of medical fields—from gynaecology, where it is used as an analgesic during childbirth, to oncology and palliative care. At present, oxycodone is one of the best-selling opioid medicines on the market.

Furthermore, oxycodone is available in various pharmaceutical forms. These range from oral formulations—where, thanks to the use of appropriate technology in application form, it is possible to prolong the drug’s duration of action and reduce its addictive potential—to parenteral forms, such as intravenous or intramuscular administration. A drawback of oral forms is that they can be altered. These forms crumble easily. As a result, people seeking a euphoric effect from oxycodone usually choose this form, only to then crush and dissolve the compound for injection or to snort the powder. Oxycodone crosses the blood–brain barrier easily and increases dopamine levels in the nucleus accumbens. This mechanism is thought to be responsible for oxycodone’s high addictive potential.

In addition, people who use oxycodone develop tolerance over time. This leads to an increase in the dose taken. There is no single toxic dose that applies to everyone; it depends on the individual’s condition, including other substances they are taking. For example, benzodiazepines significantly increase the risk of respiratory depression, the main symptom associated with an oxycodone overdose.

High sales, widespread use in the medical setting, and a growing trend towards inappropriate use, addiction and oxycodone poisoning highlight the need to find a detection method capable of identifying oxycodone both in patients being treated with this opioid and in individuals using the compound for non-medical purposes. Immunoenzymatic methods, such as ELISA, are ideal for screening as they are easy to perform, inexpensive and provide rapid results; however, due to numerous factors that can interfere with the results, a positive result should be confirmed using more sensitive methods, such as liquid chromatography/mass spectrometry. The correct diagnostic procedure should be initiated at the stage of selection of the diagnostic sample. Blood is suitable for both ante-mortem and post-mortem patients. Urine, vitreous humour and hair are also suitable. Meanwhile, oral fluid is generally collected from living patients where there is a suspicion of recent ingestion of this drug. On the other hand, vitreous humour can only be collected from patients post-mortem. Each sample type also has a different detection window—blood and oral fluid have relatively short windows, but oxycodone can be detected 24 h after ingestion; the urine detection window is up to 4 days (and, when detection is extended to include oxycodone metabolites, this is increased by a further 2 days); and in hair, oxycodone can be detected for up to around 90 days.

## Figures and Tables

**Figure 1:**
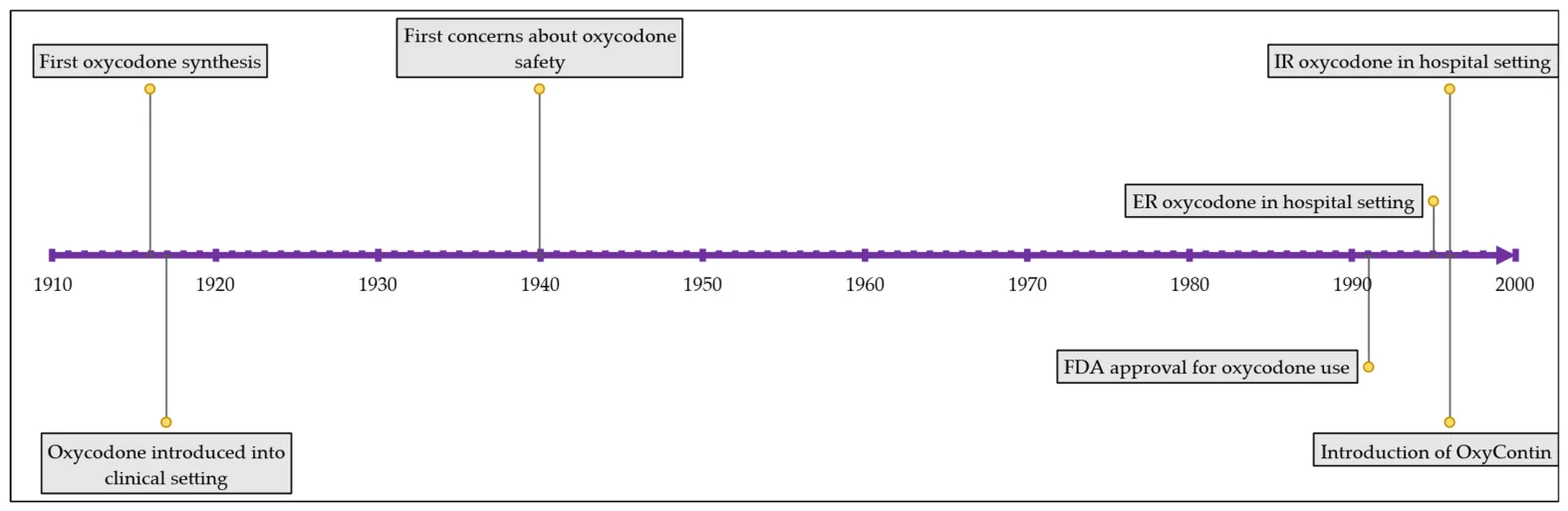
The history of oxycodone (prepared using Excel 2021).

**Figure 2:**
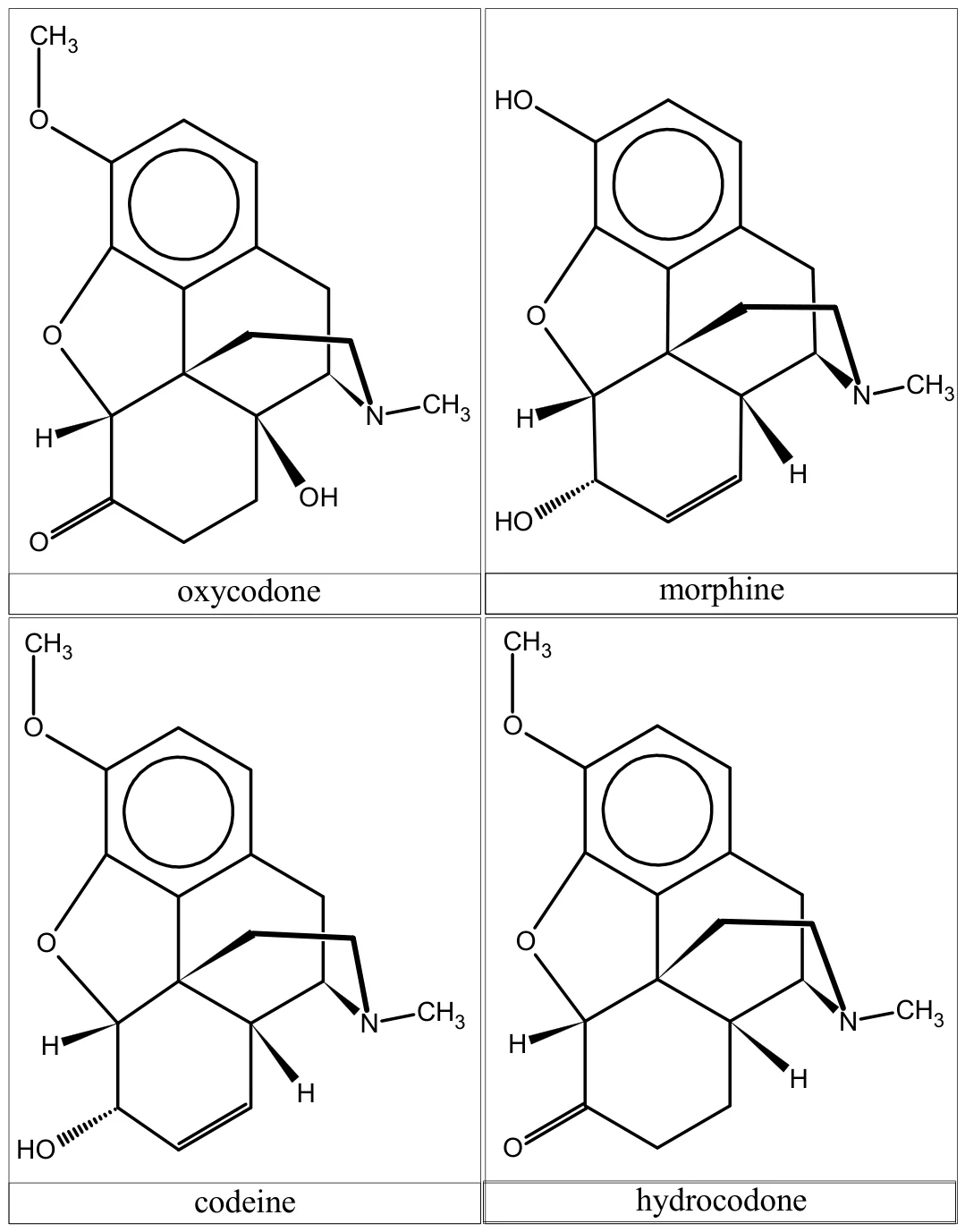
Chemical structures of oxycodone, morphine, codeine and hydrocodone (prepared using ChemSketch Version 12.01).

**Figure 3:**
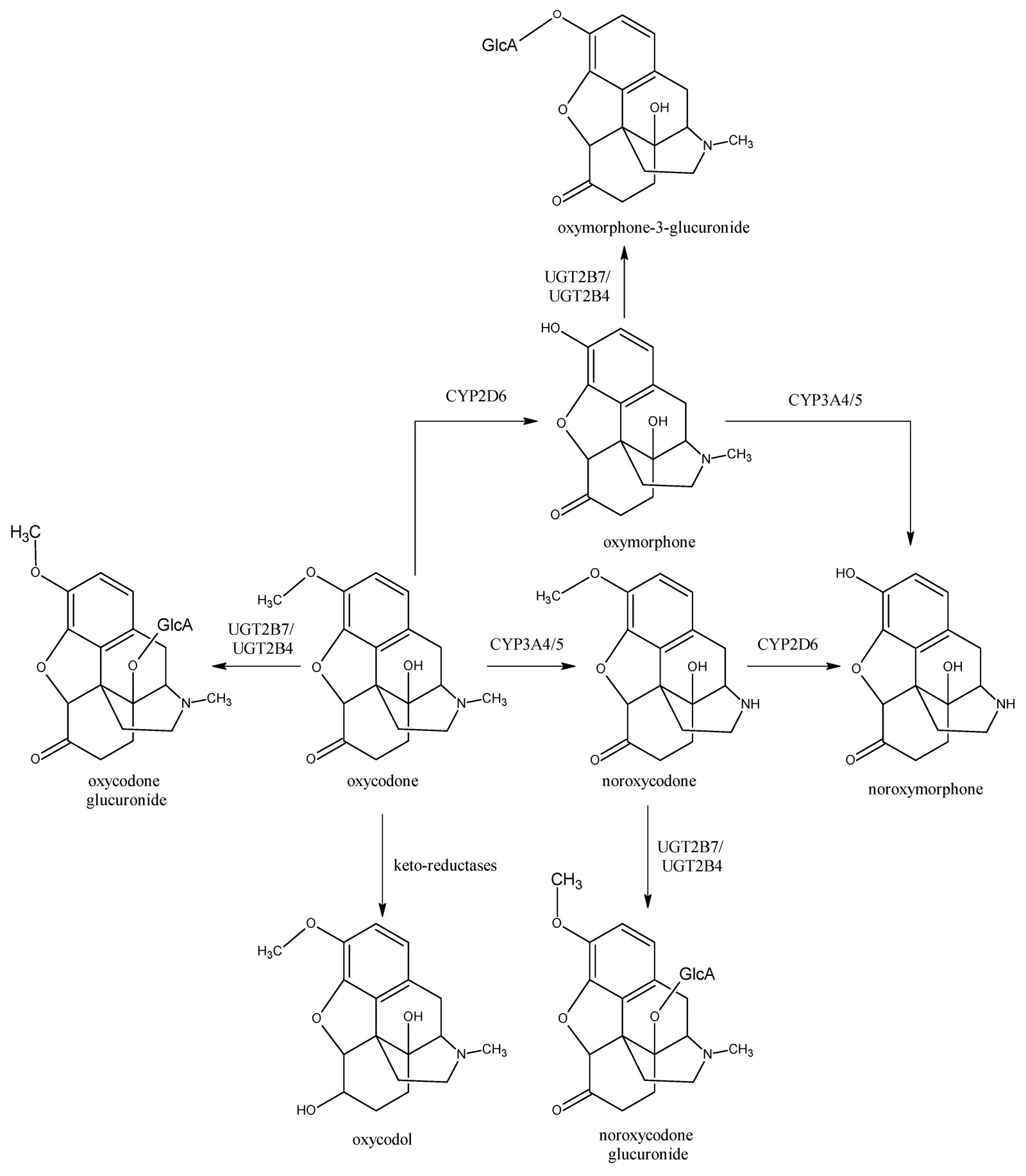
Metabolism of oxycodone; GlcA = glucuronide (prepared using ChemSketch Version 12.01).

**Figure 4:**
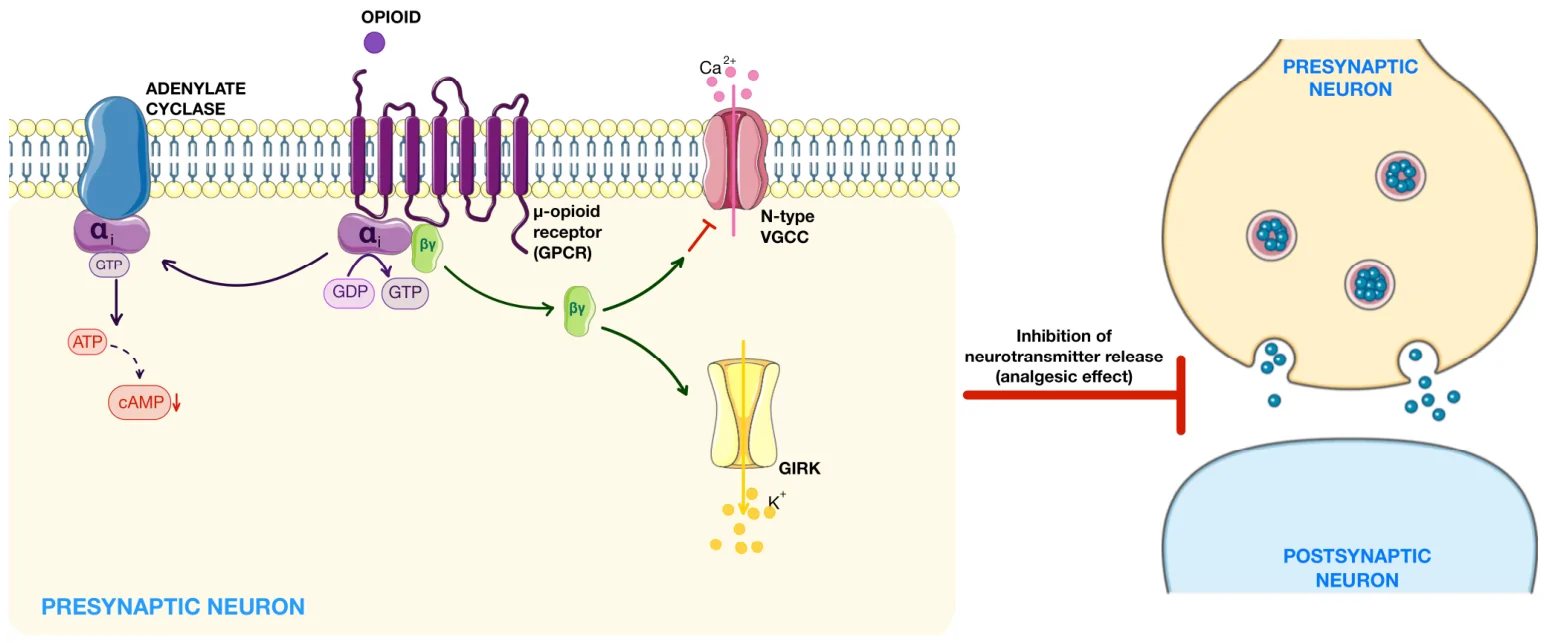
Molecular mechanism of oxycodone. Oxycodone activates the μ-opioid receptor (GPCR), leading to the dissociation of the Gαi/o and Gβγ subunits. Gαi/o inhibits adenylyl cyclase and reduces intracellular cAMP, while Gβγ inhibits voltage-gated N-type Ca^2+^ channels and activates GIRK K^+^ channels. These effects reduce neuronal excitability and neurotransmitter release, contributing to opioid-induced analgesia. Adapted from Servier Medical Art (https://smart.servier.com accessed on 10 September 2026), licenced under CC BY 4.0. Skowron, I.

**Table 1:** Comparison of different application forms of oxycodone.

Route	Bioavailability	Advantages of Route
Oral	60–87% [22]	Effective painkiller in various medical situations Reduced costs Reduced hospitalisation of patients during multimodal treatment Rare nausea
Intravenous	100%	Stabilisation of parameters during and 10 min following extubation For patients with digestive disorders
Nasal	Inter-individual variability [77]	For patients with digestive disorders
Epidural	40% enters the bloodstream, 60% enters the cerebrospinal fluid [80]	Less common side effects
Rectal	50–60% [22]	Elimination of first-pass metabolism Protection of drug degradation in stomach

**Table 2:** Comparison of LC/MS and immunoassay methods.

Feature	Chromatography	Immunoassay
**Examples of methods**	LC/MS; LC-MS/MS; GC/MS; HRMS; UHPLC-MS/MS	ELISA, EMIT, KIMS, cloned enzyme donor immunoassay
**Sensitivity**	High	Dependent on test
**Selectivity**	High	Dependent on test
**LOQ**	Dependent on test (LC-MS/MS: 0.5 ng/mL [189] UHPLC-MS/MS: 0.02 ng/mL [194])	Dependent on test (ELISA: 1 ng/mL [206])
**Ability to distinguish parent drug from metabolite**	Yes	Dependent on test (rather not)
**Cost**	High	Low
**Time to obtain results**	Long	Short
**Necessary personnel**	Qualified and trained	No special requirements
**Interfering substances**	Matrix effects	Dependent on test (other opioids [187], poppy seed metabolites [205], quinolones [208])
**Role**	Confirmatory	Screening

**Abbreviations:** LC/MS—liquid chromatography coupled with mass spectroscopy; LC-MS/MS—liquid chromatography coupled with tandem mass spectroscopy, GC/MS—gas chromatography coupled with mass spectroscopy; HRMS—high-resolution mass spectroscopy; UHPLC-MS/MS—Ultra-High-Performance Liquid Chromatography coupled with tandem mass spectrometry; ELISA—enzyme-linked immunosorbent assay; EMIT—enzyme-multiplied immunoassay technique; KIMS—kinetic interactions of microparticles in solution; LOQ—limit of quantification.

**Table 3:** Comparison of various types of samples.

Diagnostic Material	Sampling Site	Window of Detection	Incursion of Privacy of Ante-Mortem Patient	Stability During Storage	Interferences	References
**Blood**	Ante-mortem: the antecubital vein Post-mortem: femoral vein, heart chambers	0.5–24 h	Invasive	In fridge or freezer: 4 °C—1 month −20 °C—6 months	Post-mortem redistribution Conditions altering body water content, e.g. burns, injuries, blood loss	[221,243]
**Dried blood spot**	Infant: heel vein	0.5–24 h	Low	Dry place: Room temperature—1 month	Blood conditions	[243]
**Urine**	Urinary tract (urine stream, catheter, directly from urinary bladder)	1 h–4 days	High	In fridge or freezer: 4 °C—1 month −20 °C—6 months	Not enough time between intake and death Deliberate contamination	[210,221,229]
**Vitreous humour**	Eye	Unspecified due to properties of the material	Not applicable (only from post-mortem patients)	In fridge or freezer: −20 °C—year	Low correlation between blood and vitreous humour	[233]
**Oral Fluid**	Oral cavity	15 min–2 days	Low	In fridge or freezer: Longer than blood −70 °C—22 months	Low correlation between blood and oral fluid Oral intake Smoking Age, gender, diet and health status	[237,243]
**Hair**	Body hair (preferably the vertex)	10–90 days	Low	Room temperature: Indefinitely	Contamination Not enough time between intake and death Melanin External factors (heat, sunlight, water)	[246,249]
**Nails**	Fingers and toes	5–14 months	Low	Room temperature: Indefinitely	External contamination	[253,254]

## Data Availability

All data discussed derive from the cited publications.

## Abbreviations

The following abbreviations are used in this manuscript:

ADF	Abuse-deterrent formulation
CNS	Central nervous system
CR	Controlled release
DBS	Dried blood spot
DOR	Delta opioid receptor
ELISA	Enzyme-linked immunosorbent assay
EMIT	Enzyme-multiplied immunoassay technique
ER	Extended release
FDA	Food and Drug Administration
GABA	Gamma-aminobutyric acid
GC/MS	Gas chromatography–mass spectrometry
GIRK	G Protein-Gated Inwardly Rectifying Potassium
GPCRs	G protein-coupled receptors
HCV	Hepatitis C virus
HRMS	High-resolution mass spectrometry
IMF	Illicitly manufactured fentanyl
IR	Immediate release
KIMS	Kinetic interactions of microparticles in solution
KOR	Kappa opioid receptor
LC/MS	Liquid chromatography–mass spectrometry
LC-MS/MS	Liquid chromatography–tandem mass spectrometry
LOQ	Limit of quantification
LLOQ	Low level of quantification
MOR, MOP	Mu opioid receptor
PWID	People Who Inject Drugs
SDP	Single-dose product
TLR4	Toll-like receptor 4
UHPLC-MS/MS	Ultra-High-Performance Liquid Chromatography coupled with tandem mass spectrometry
UGT	Uridine diphosphoglucuronosyltransferase
USA	United States of America
